# Supplementary material for: Balancing between being the most valuable player (MVP) and passing the ball: a qualitative study of support when living with chronic pain in Sweden
Source: BMJ Open. 2024 Jan 31;14(1):e079229. doi: 10.1136/bmjopen-2023-079229 (PMC10831426; doi:10.1136/bmjopen-2023-079229)
Supplement: Supplementary data [file bmjopen-2023-079229supp001.pdf]

Pre-understanding

I believe persons with chronic pain often feel as if they have been treated poorly in encounters with healthcare professionals. I think they perceive that their pain is not taken seriously and that they are not being believed. I also think they perceive they are not being given the required help and struggle to get help. I believe they mostly have been in contact with medical doctors.

I believe they experience that their social network is not being invited to participate in their care. I think they feel support from their social network without including the network within their care. I do not know how they perceive support, but I can imagine that the employer, family, and friends are significant and their relationships are often impacted by pain. People with chronic pain want to involve their social network if healthcare professionals ask them.
