# Supplementary material for: Balancing between being the most valuable player (MVP) and passing the ball: a qualitative study of support when living with chronic pain in Sweden
Source: BMJ Open. 2024 Jan 31;14(1):e079229. doi: 10.1136/bmjopen-2023-079229 (PMC10831426; doi:10.1136/bmjopen-2023-079229)
Supplement: Supplementary data [file bmjopen-2023-079229supp002.pdf]

Interview guide

The aim is to elucidate the meaning of lived experiences of support from the social network and healthcare sector in persons with chronic pain.

Research questions

How do persons with chronic pain experience support from the healthcare sector?

How do persons with chronic pain experience support from their social network outside the healthcare sector?

How do persons with chronic pain perceive involving their social network in their care and rehabilitation?

Support within the healthcare sector

Do you want to tell me when you first contacted the healthcare sector regarding your pain?

How have you perceived the help you've gotten from the healthcare sector since then?

- What did you feel when you did/did not receive that help?
- How has the help you've received from the healthcare sector made a difference to you?

What kind of help do you feel you would have liked to have but have not gotten from the healthcare sector?

- Are there any symptoms you would have liked to receive more help with?
- How did you feel when you received/did not receive that help?

How would you like the healthcare sector to help you?

Support from the social network

Which persons outside of the healthcare sector have been important to you regarding your pain?

- How has contact with those people helped you?
- What kind of difference has that contact made for you?

Are there others outside the healthcare sector who have been important to you regarding your pain?

- How has contact with these people helped you?
- What difference has the contact with these persons made for you?

Are there any persons outside the healthcare sector you would have liked to receive more support from?

- Do you want to tell me more about this?
- What did you feel when you did not receive that support?

Involvement of the social network in care

How have persons from outside the healthcare sector been involved in your care? (for instance, next of kin).

- In which parts of the care have they been involved?
- How did it come about that they got involved?

- Who initiated the involvement?

How has it worked to have them involved in your care?

- How have you experienced it?

How do you feel about involving persons outside of the healthcare sector in your care?

- In which situations would you like to involve persons from outside the healthcare sector in your care?

What would facilitate involving other persons in your care?

How do you perceive the healthcare sector's attitude towards involving other persons?

Concluding

Is there anything that we've discussed that you would like to talk more about or something we haven't addressed that you would like to add?
