# Supplementary material for: Balancing between being the most valuable player (MVP) and passing the ball: a qualitative study of support when living with chronic pain in Sweden
Source: BMJ Open. 2024 Jan 31;14(1):e079229. doi: 10.1136/bmjopen-2023-079229 (PMC10831426; doi:10.1136/bmjopen-2023-079229)
Supplement: Author's manuscript [file bmjopen-2023-079229.draft_revisions.pdf]

BMJ Open is committed to open peer review. As part of this commitment we make the peer review history of every article we publish publicly available.

When an article is published we post the peer reviewers' comments and the authors' responses online. We also post the versions of the paper that were used during peer review. These are the versions that the peer review comments apply to.

The versions of the paper that follow are the versions that were submitted during the peer review process. They are not the versions of record or the final published versions. They should not be cited or distributed as the published version of this manuscript.

BMJ Open is an open access journal and the full, final, typeset and author-corrected version of record of the manuscript is available on our site with no access controls, subscription charges or pay-per-view fees (http://bmjopen.bmj.com).

If you have any questions on BMJ Open's open peer review process please email info.bmjopen@bmj.com

BMJ Open

Balancing between being the Most Valuable Player (MVP) and passing the ball: A qualitative study of support when living with chronic pain

Journal:	*BMJ Open*
Manuscript ID	bmjopen-2023-079229
Article Type:	Original research
Date Submitted by the Author:	25-Aug-2023

Complete List of Authors:	Lilja, Veronica; University of Gothenburg Institute of Health and Care Sciences, Sahlgrenska Academy; University of Gothenburg Centre for Person-Centred Care, Sahlgrenska Academy<br>Wallström, Sara; University of Gothenburg Institute of Health and Care Sciences, Sahlgrenska Academy; University of Gothenburg Centre for Person-Centred Care, Sahlgrenska Academy<br>Saarijärvi, Markus; Karolinska Institute Department of Neurobiology Care Sciences and Society, Division of Nursing; University of Gothenburg Centre for Person-Centred Care, Sahlgrenska Academy<br>Lundberg, Mari; Sophiahemmet University, Department of Health Promoting Science; University of Gothenburg Centre for Person-Centred Care, Sahlgrenska Academy<br>Segertoft, Vivi-Anne; Personskadeförbundet,<br>Ekman, Inger; University of Gothenburg Institute of Health and Care Sciences; University of Gothenburg Centre for Person-Centred Care

Keywords:	Chronic Pain, Social Support, QUALITATIVE RESEARCH, PAIN MANAGEMENT

SCHOLARONE™
Manuscripts

I, the Submitting Author has the right to grant and does grant on behalf of all authors of the Work (as defined in the below author licence), an exclusive licence and/or a non-exclusive licence for contributions from authors who are: i) UK Crown employees; ii) where BMJ has agreed a CC-BY licence shall apply, and/or iii) in accordance with the terms applicable for US Federal Government officers or employees acting as part of their official duties; on a worldwide, perpetual, irrevocable, royalty-free basis to BMJ Publishing Group Ltd ("BMJ") its licensees and where the relevant Journal is co-owned by BMJ to the co-owners of the Journal, to publish the Work in this journal and any other BMJ products and to exploit all rights, as set out in our licence.

The Submitting Author accepts and understands that any supply made under these terms is made by BMJ to the Submitting Author unless you are acting as an employee on behalf of your employer or a postgraduate student of an affiliated institution which is paying any applicable article publishing charge ("APC") for Open Access articles. Where the Submitting Author wishes to make the Work available on an Open Access basis (and intends to pay the relevant APC), the terms of reuse of such Open Access shall be governed by a Creative Commons licence – details of these licences and which Creative Commons licence will apply to this Work are set out in our licence referred to above.

Other than as permitted in any relevant BMJ Author's Self Archiving Policies, I confirm this Work has not been accepted for publication elsewhere, is not being considered for publication elsewhere and does not duplicate material already published. I confirm all authors consent to publication of this Work and authorise the granting of this licence.

For peer review only

Balancing between being the Most Valuable Player (MVP) and passing the ball: A qualitative study of support when living with chronic pain

Veronica Lilja, corresponding author (Orcid ID 0000-0002-5531-6358)

- Institute of Health and Care Sciences, Sahlgrenska Academy, University of Gothenburg, Gothenburg, Sweden
- University of Gothenburg Centre for Person-Centred Care (GPCC), Sahlgrenska Academy, University of Gothenburg, Gothenburg, Sweden

Sara Wallström: Orcid ID:0000-0001-7579-4974

- Institute of Health and Care Sciences, Sahlgrenska Academy, University of Gothenburg, Gothenburg, Sweden
- University of Gothenburg Centre for Person-Centred Care (GPCC), Sahlgrenska Academy, University of Gothenburg, Gothenburg, Sweden

Markus Saarijärvi: Orcid ID: 0000-0002-7053-6930

- Division of Nursing, Department of Neurobiology, Care Sciences and Society, Karolinska Institutet, Stockholm, Sweden
- University of Gothenburg Centre for Person-Centred Care (GPCC), Sahlgrenska Academy, University of Gothenburg, Gothenburg, Sweden

For peer review only

Mari Lundberg: Orcid ID 0000-0001-7123-0362

- Department of Health Promoting Science, Sophiahemmet University, Stockholm, Sweden
- University of Gothenburg Centre for Person-Centred Care (GPCC), Sahlgrenska Academy, University of Gothenburg, Gothenburg, Sweden

Vivi-Anne Segertoft: Orcid ID 0009-0009-7916-668X

- The Swedish Association for Survivors of Accident and Injury, Uddevalla, Sweden

Inger Ekman: Orcid ID: 0000-0002-5559-5203

- Institute of Health and Care Sciences, Sahlgrenska Academy, University of Gothenburg, Gothenburg, Sweden
- University of Gothenburg Centre for Person-Centred Care (GPCC), Sahlgrenska Academy, University of Gothenburg, Gothenburg, Sweden

For peer review only

ABSTRACT

Objective: This study aimed to elucidate the meaning of lived experiences of support from the social networks and healthcare sector in persons with chronic pain.

Design: A qualitative, phenomenological hermeneutic method was used to analyse interview data.

Setting: Participants were recruited from patient organizations in Sweden.

Participants: Ten (seven women, two men and one non-binary) individuals with chronic musculoskeletal pain were included.

Findings: The meaning of lived experiences of support in persons with chronic pain involves balancing between being the most valuable player (MVP) and passing the ball, meaning balancing between being a capable person and accepting support to be that capable person.

Conclusion: For persons with chronic pain, support means balancing between being capable (the MVP) and willing to accept support (passing the ball), which aligns with person-centred care. Policymakers, managers, and clinical professionals should consider our findings when planning and implementing care for persons with chronic pain. Future research should focus on how the healthcare sector can create support to enable persons with chronic pain to be the most valuable player (MVP) while being able to pass the ball in their social networks and healthcare sector.

Keywords: chronic pain, social support, qualitative research, pain management

STRENGTHS AND LIMITATIONS

- Chronic pain affects many people worldwide and understanding the meaning of healthcare sector and social network support is vital to provide tailored assistance and practical solutions.

- Further insights was achieved with a patient representative who actively participated in the analysis and manuscript process.

- Using a metaphor to describe the findings created a new understanding of the meaning of support

For peer review only

when living with chronic pain.

- A diversity of basic demographic indicators (age, geographic location, and occupational status) is a strength of the study.

- A limitation is that most participants were well-educated, female, and born in Sweden.

For peer review only

INTRODUCTION

Pain is defined as "an unpleasant sensory and emotional experience associated with, or resembling that associated with, actual or potential tissue damage" (1). Chronic pain persists or recurs for over 3 months (2) and is viewed as a disease, not just a symptom. The prevalence of chronic pain differs between studies, contexts, and types of measurement. In a large European study comprising 16 countries the prevalence of chronic pain was estimated to be 19% (3). A US study showed a similar prevalence rate (20.4%) (3).

Persons with chronic pain often struggle with comorbidities (e.g., depression, anxiety, cardiovascular disease, and cancer) (4), side effects from medication (5), and poor health-related quality of life (6). Chronic pain also tends to adversely affect sleep, daily activities, relationships, and the ability to work (5). Suffering from pain is often perceived as invisible to others, which can contribute to feeling unjustly treated in society (7). From a societal and health economic perspective, chronic pain presents challenges because it is a common reason for sick leave (8-10) and healthcare-seeking behavior (4, 5, 9). In Sweden, the cost (indirect and direct) of chronic pain-related diagnoses was estimated at 32 billion euros per year in 2012; 59% were due to sick leave and early retirement (11).

A meta-synthesis showed that support from family and friends is important in pain management (12). Social support can include sharing advice, expressions of empathy and contributing to positive feelings (13, 14). In contrast, lacking support can lead to feelings of loneliness and not being needed (15). Peer support interventions have been shown to decrease pain severity and interference (16). However, there is conflicting evidence of the positive effects of support. A peer support intervention for veterans with musculoskeletal pain found no statistically significant impact on pain (17). Studies investigating spouses'

For peer review only

participation in educational interventions suggest no additional benefits of including a partner (18) and that participating with a partner could make participants more prone to fatigue and lower self-efficacy compared to not participating with a partner (19). Further explorative research on support is needed.

Collaborative relationships with healthcare professionals constitute a support that seems to facilitate self-management of pain (12). Chronic pain is complex and the biopsychosocial model, involving biological, psychological, and social factors, has been successfully used in pain management (20). Evidence-based practice is based on a multimodal approach, including a healthy lifestyle, physiotherapy, and pharmacological and psychological treatment (21). There is also some evidence for the benefits of complementary therapies (21). Moreover, research has shown that persons with chronic pain desire better support from the healthcare sector (22) and feel that healthcare professionals rarely take their condition seriously (8, 15, 22).

Because of conflicting evidence and the complexity of support for persons with chronic pain, there is a need to understand the meaning of support, both within and outside the healthcare system. A deeper understanding of the phenomenon would facilitate the comprehension of the need for support and could aid in shedding light on what kind of support persons with chronic pain want and need. In addition, this understanding can help in developing tailored interventions for this patient group.

Therefore, this study aims to elucidate the meaning of lived experience of support from social networks and the healthcare sector in persons with chronic pain.

For peer review only

METHODS

Design

The present study applied a qualitative method with a phenomenological hermeneutic approach inspired by Lindseth and Norberg (23). Phenomenological hermeneutics is suitable for interpreting the essential meaning of a lived phenomenon through text narratives (23). The present study follows Standards for Reporting Qualitative Research guidelines (24).

Participants and setting

Participants were recruited from four Swedish patient organizations by a Facebook post or an e-mail sent from the organisations. Combined, the organisations have over 47 thousand members, making them a good point of contact. Persons interested in participating in the study contacted the first author (VL) by e-mail. Inclusion criteria were ≥18 years of age, living in Sweden, and having chronic musculoskeletal pain (defined as "chronic pain arising from musculoskeletal structures" (25)). Persons who primarily seemed to struggle with other conditions, such as concurrent cancer diagnosis, were excluded. Participants who mainly wanted to share their narratives about musculoskeletal pain but had previously undergone cancer treatment or had another pain-related diagnosis were not excluded.

Some 177 persons (1 non-binary, 4 men, 172 women) expressed interest in participating. A purposive sampling strategy was employed to include participants from different parts of Sweden, regardless of treatment or current contact with healthcare. Participants with vast experience of support and willingness to share were purposefully selected, as this allows for rich data to be achieved (26). As maximum variation sampling allows uncovering common meaning across demographic differences (26), a diversity of experiences of support, age,

For peer review only

geographic location, sick leave rate, and background diagnosis was strived for even if most potential participants were women. Eight participants were initially included, and their narratives were deemed sufficient to answer the study's research question. However, another two participants were interviewed to achieve greater variation in educational level. None of the participants declined to participate. The material was then deemed sufficiently rich, and after discussions within the research group, inclusion was halted.

The author conducting the interviews (VL) has a nursing and public health background. Before the study, the interviewer's pre-understanding was written (see supplemental file 1) and was reflected upon in the analysis. VL was a novice in phenomenological hermeneutics; however, her lack of experience in this area was complemented by the research group's extensive experience with this method. A patient representative was also part of the research group and contributed with experience of living with chronic pain.

Data collection

A semi-structured interview guide (supplemental file 2), derived from Brinkmann and Kvale (27), was developed by VL with input from three other authors (SW, ML, IE). The guide covered three domains of support: the healthcare sector, social networks, and how support from social networks could be integrated within care. The interview guide contained open-ended questions with suggestions for probing questions. The interview guide was piloted in the first two interviews and slightly revised. The narratives from the two pilot interviews were deemed relevant and thus included in the data analysis. Seven interviews were conducted digitally through Zoom™, one by phone, and two face-to-face between February 2021 and August 2022. The interviews were recorded and transcribed verbatim by VL.

For peer review only

Participants chose the date, place, and format for the interviews. Both video and telephone interviews are trustworthy alternatives to face-to-face interviews in qualitative research (28).

Patient and public involvement

Four patient organizations contributed to recruiting study participants. One of the co-authors (VS) is a patient representative.

Ethical considerations

Ethical approval for this study was granted by the Swedish Ethical Review Authority (Reg nr 2020-02491). In addition, the study was conducted according to the ethical principles outlined in the Declaration of Helsinki (29). All participants received written and verbal information about the study. Written and verbal informed consent was obtained from all participants and all data were handled with strict confidentially. Participants' identities were removed during data transcripts. Audio recordings, transcripts, and a keycode were safely stored in different locations to protect the participants' identity and personal information.

Data analysis

The data were analysed with phenomenological hermeneutics. The method, influenced by Ricoeur's theory of interpretation and developed by Lindseth and Norberg (23), involves three intertwined phases: *naïve understanding*, *structural analysis,* and *comprehensive understanding*. Through the hermeneutic spiral, the phases are constantly overlapping,

For peer review only

revisited and compared to each other to move between explanation and understanding by interpretation of the whole and the parts.

Each interview was read several times. VL then formulated a naïve understanding for each interview before cultivating a common naïve understanding. The structural analysis was performed with the software NVivo version 12 by VL with input from the other authors. The text of each interview was divided into meaning units and then coded. All text was considered, but only text associated with the study's aim was included in the structural analysis. The codes were continuously compared to the naïve understanding. The interviews were read through again, and the naïve understanding was revised and compared to the structural analysis. This process was repeated several times. Eventually, tentative themes and sub-themes were formulated and compared to the codes and the naïve understanding of the interviews. VL and IE constantly discussed and reformulated the tentative findings before consulting the other authors.

Table 1. Examples of structural analysis.

Text	Code	Sub-theme	Theme	Main theme
The appreciation of myself was destroyed already at the beginning of my sickness … and the value… my own value. Because they did not see me as competent. They saw only my illness. So, this has been awfully hard. And there is still frustration in not getting	Self-worth	Being a valuable player and not only being the injured one.	Being the MVP	Balancing between being the MVP and passing the ball in a match against pain

For peer review only - http://bmjopen.bmj.com/site/about/guidelines.xhtml

this kind of recognition.				
I was so incredibly fortunate; he is a great guy, a great doctor and, and... he supports me in the next step.	Someone fights with you	Being able to pass the ball when you have to	Passing the ball	Balancing between being the MVP and passing the ball in a match against pain

In the comprehensive interpretation VL and IE compared the pre-understanding, the naïve understanding, and the structural analyses to the existing literature. The comprehensive understanding was then discussed between all authors. MS, SW, and VS read and gave feedback on the findings. ML read all the interviews and the results to ensure the interpretations were reasonable.

FINDINGS

Ten participants were included in the study. The demographic characteristics of participants are described in Table 2. The interviews lasted between 39 and 101 minutes (mean 77 minutes).

Table 2. Participant characteristics.

**Characteristics**	
**Age**	
Mean (range)	48 years (24-70)
**Sex**	

For peer review only

Female	7
Male	2
Non-binary	1
**Place of birth**	
Born in Sweden	9
Not born in Sweden	1
**Diagnosis**	
Fibromyalgia	1
Post-polio syndrome	2
Spinal injury	1
Ehlers-Danlos Syndrome (hEDS and HSD)	4
Fibromyalgia and Ehlers-Danlos Syndrome	2
**Duration of pain**	
Mean (range)	32.6 years (14-66)
**Educational level**	
High school degree	3
University degree	7
**Occupational status**	
Working full-time	5
Working part-time due to sick leave	3

For peer review only

Unemployed without financial support	1
On disability pension	1
**Relationship status**	
Partner	7
Single	3

Findings from the analyses are presented in the following order: First, the naïve understanding is given, then the structural analyses, and finally, a comprehensive interpretation of these parts together with the pre-understanding.

Naïve understanding

The naïve understanding of the meaning of support is that it reinforces the participants' ability to manage their pain and everyday life. The participants seek to address their pain and life situation independently but need support to achieve this goal. They feel lonely in and diminished by the healthcare sector, often seen as unavailable or hostile. Support from the right healthcare professional, someone who listens and will go the extra mile to establish a diagnosis and provide help, makes a big difference in the participants' perception of their capability. Experiences of support from outside the healthcare sector vary considerably. Although the participants are eager to manage on their own, social networks that believe in them, show compassion and fight together with them are essential.

For peer review only

Structural analyses

The main theme, themes, and sub-themes are described below and in Table 3.

Balancing between being the Most Valuable Player (MVP) and passing the ball in the match against pain

Living with chronic pain can be a constant battle, and just like a football match, it can vary in intensity. In these analyses we have used the metaphor of a football match. In this football match pain is the opponent, the person with chronic pain is the MVP (Most Valuable Player), and the teammates are persons within the MVP's social networks and healthcare sector. The social networks could include family, partners, friends, employers, colleagues, peers with chronic pain, personal trainers, personal coaches, persons performing complementary therapies, neighbours and pets. The ball (interpreted as pain management) in this football match is passed around to members of the MVP's team to win the match against pain. The attempt to win the match does not mean being pain-free but living the life the person with chronic pain wants to live despite the pain. The meaning of lived experiences of support refer to the constant balance between managing alone (being the MVP) and accepting help from others (passing the ball), which is further explained through the themes and sub-themes.

Table 3. Overview of the main theme, themes, and sub-themes.

**Main theme: Balancing between being The Most Valuable Player (MVP) and passing the ball in the match against pain**	
Theme: Being the MVP	Theme: Passing the ball

For peer review only

Sub-theme: Being a valuable player and not just the injured one	*Sub-theme: Being part of a team*
Sub-theme: Being trusted to dribble	*Sub-theme: Being able to pass the ball when you have to*
	Sub-theme: Needing a substitute without being a benchwarmer

Being the MVP

The participants wanted to contribute to society just like everyone else and manage independently by taking the lead in their care and daily life. They also desired to be who they were without the pain dominating their lives. In football terms they aspired to be the MVP in all aspects of their lives. Being believed and listened to were important aspects of being trusted to dribble the ball, which is essential when seeking to be the MVP.

Being a valuable player and not just the injured one

The participants sought to be recognized foremost as the persons they were, with unique personalities and experiences. When perceived as a product of their pain, they felt excluded and viewed as someone who could not accomplish much. When the social networks provided support by accepting the pain as part of the participants but still recognising them for who they were and their capabilities, it facilitated their acceptance of themselves and the pain. Pets could also contribute to this kind of support, as they were indifferent to the participants' pain and provided unconditional love and companionship. Participants also wanted healthcare professionals to recognize them as the persons they were. They felt

For peer review only

supported when professionals aimed at strengthening their resources by not only focusing on their limitations but also on their abilities. The participants felt an enhanced power by gaining access to self-help devices (e.g., orthoses).

"It is about being an important part of society, to contribute… instead of being the one others should take care of. Self-help devices help to achieve that."

- Participant 10.

The social networks provided support by requesting the participants' help and advice. This support reinforced the participants' view of themselves as unique, capable, meaningful, and contributors rather than just someone with pain. Providing peer support to others with pain within a patient organization exemplifies how the participants contributed. Being recognized as a person, rather than the pain the individual struggles with, could be interpreted as being recognized as a valuable player in football. As a valuable football player, the injury will not matter, as everyone appreciates the player’s efforts on the field and knows their potential.

Being trusted to dribble

To the participants a diagnosis was important to get their experiences acknowledged, understanding their pain and being believed. A diagnosis meant validating their condition and was also experienced as facilitating being believed, trusted, and understood by the social networks. When the expectations from others did not clash with their abilities while still being trusted with tasks they could perform, the participants' view of themselves as capable persons was reinforced.

For peer review only

The participants sometimes felt they received better support within complementary therapies compared to traditional care. They felt listened to and perceived as capable persons. This complimentary support, however, was not always affordable because of the participants' often strained economy. Being believed and listened to when sharing experiences of pain could be interpreted as a football player being trusted by other teammates. The teammates show that the player and their abilities are trusted by passing them the ball and allowing them to dribble.

"To receive understanding for my experience of pain, that every individual experiences pain differently, and that it [the pain] is taken seriously is a crucial factor to feel that you're getting support."

- Participant 1.

Passing the ball

Living with pain was challenging, and occasionally the participants felt their abilities were inadequate to engage with their situation. They needed a team to help them regain trust in themselves and fight to improve their situation. In football the MVP must have a team to pass the ball to, as they cannot win the game alone.

Being part of a team

The participants often felt alone struggling with pain. Navigating the healthcare system and the social insurance agency's policies was difficult. Fighting different systems alone evoked feelings of being diminished, vulnerable, and powerless. When they found regular contact with a healthcare professional willing to help and possibly involve other professionals, or

For peer review only

when their networks was ready to help them fight the system, they did not feel as lonely. This regular contact enhanced the participants' sense of capability. Having support from others, they felt stronger and could accomplish more.

Reducing loneliness by accepting support from others could be analogous to being part of a football team. The individual is stronger with teammates and stands a better chance of beating the other team (i.e., overcoming chronic pain).

"We [participant and health care team] will do what we can to solve what we can together. I also have to do my part, but we converge in that understanding that there is a problem, and we will try to solve it together— teamwork."

- Participant 8.

Being able to pass the ball when you have to

Participants felt hopeless when told by health care professionals that there was nothing left to be done after undergoing several medical treatments or when test results failed to reveal the cause of their pain. The hope of being capable persons was reinstated when they were provided support (e.g., tailored training programs or self-help devices). When social networks provided support by facilitating daily tasks, tailored work schedules, ideas for pain management, or contacts with trusted health care professionals, the participants felt hope that their circumstances would improve, and they could live a desired everyday life.
Regaining hope could be interpreted as having someone to pass the ball to when the football player can no longer dribble on their own (when the pain is unbearable, and they do not know how to move on). By passing the ball, the hope remains that the team can control

For peer review only

the ball instead of losing it to the other team.

"When I get this lumbago, I believe it will never pass, and I have to live my whole life like this. And then I talk to him [partner], and he reminds me it will pass and be all right. It is good to be reminded. Otherwise, I'll go into that tunnel, thinking everything will go bad."

- Participant 4.

Needing a substitute without being a benchwarmer

The participants considered it challenging to determine what they could share about their situation with their networks (particularly family and friends) without appearing as a burden. Not wanting to strain their networks or cause worry discouraged the participants from including them in their care. Some healthcare professionals blamed the participants for their situation, whereas others went beyond their duties to support the participants by writing certificates during their free time (e.g., lunch break). That kind of support made a huge difference, as the participants needed the certificates to apply for services and sick leave. At the same time, the feeling of being a burden was enhanced because the healthcare professionals had to sacrifice their spare time to offer that support. Fear of being a burden could be seen as fear of losing the title of MVP and becoming a benchwarmer, i.e., slowing down the team.

"But some [healthcare professionals] set aside that time and try their best within the system they've been put in. It comes with a price for them, but it is significant that they give me that

For peer review only

reception and set aside time to listen."

- Participant 5.

Interpretation of the whole

As the main theme suggests, the meaning of the participants' lived experiences of support from the healthcare sector and networks is to balance being a capable person and accepting help from others to continue being that capable person. Using football terms, the MVP and the team compete against pain (see Figure 1).

All people want to feel needed and useful. Any football player's dream is to be the MVP whose actions ultimately determine the match's outcome. Persons with chronic pain are no different, i.e., they aspire to be important. However, not even the MVP can win a match without team support. Many football players (especially injured players) will not play during the full 90 minutes but may substitute in the second half of the game without making them any less valuable to the team. They are not seen as a burden and can still be the MVP. Everyone recognizes those football players' previous efforts and potential. The teammates will pass them the ball, let the MVP dribble and await the MVP to pass it back. The organisation will provide the best available medical help and tailored training programs to help those players reach their full potential in every match. However, if you do not pass these players the ball or ensure they can pass to you when they are blocked, you've shifted over to the opposite team. When the social networks and healthcare professionals do not listen, believe them, see their capabilities, or claim that the persons with chronic pain can no longer be helped, they step over to the opposite team. The MVP might also end up on the opposing team by for instance engaging in negative thoughts and behaviours. How will

For peer review only

the MVP win the match against pain when the teammates or they themselves become the enemy? The field must be just and equitable to ensure the teammates and the MVP are on the same team fighting against the same opponent. Healthcare professionals must have fair conditions to stay on the right team and support the MVP. As with any successful football team, the team needs its MVP, and the MVP needs the team. The MVP's capability needs to be recognized within a reliable and supportive team for the MVP to perform well. The participants are the most influential persons in their own lives, and they need to be trusted as a valuable team member. People with pain feel valued and supported when the healthcare sector and social networks strengthen their abilities and capability.

[INSERT FIGURE 1 HERE]

Legend: Figure 1. The match against pain. Most Valuable Player/MVP (person with chronic pain) with the ball (pain management), the team (examples of teammates), the opponent (pain), the referee (representing fair conditions), the stadium (life), and the trophy (symbolizing the aspiration for a good life despite the pain).

DISCUSSION

This study applied an interpretative approach to explaining the meaning of support from the healthcare sector and social networks of persons with chronic pain. The findings indicate

For peer review only

that, regardless of who is providing the support, the meaning of support when living with chronic pain is to strengthen the individual's capability and, when the abilities do not seem enough, feel that someone is fighting together with the individual to regain capability. Previous studies have mainly been analysed descriptively, focusing on pain management rather than on the meaning of support (13-15). Holtrop et al. (14) found three primary purposes of important relationships in pain management: providing instrumental support, offering inspiration and motivation, and assisting in decision making. Similar results were found in our study, but our findings show that support strengthens the participants' capability. In line with the present study Holtrop et al. (14) found that persons with chronic pain wanted to be recognized as persons rather than their conditions and that their lives should be seen as no different from others. Meanwhile, they wanted their limitations due to pain to be accepted. Our study also shows that pets could provide this support, which aligns with Bair et al. (13), who found that pets can be powerful motivators in pain management. The Bair et al. (13) participants relied on support from care managers. Similarly, the findings in our study show that the meaning of support was to have someone who fights for and with the person with chronic pain when their abilities are inadequate.

The definition of complementary therapies is not universally agreed upon (30). In this study therapies not provided by the healthcare sector and complement regular care are considered complementary. Except for mindfulness, all therapies mentioned by the participants are listed by Wieland et al. (30) as complementary. Our findings suggest that complementary therapies can provide hope when regular care fails. This result concurs with Hsu et al. (31), who found that people with pain who use complementary therapies often have low expectations but hope they will provide pain relief and improved function, fitness, and well-being.

For peer review only

Acknowledging the patient as an expert and capable person is fundamental to person-centred care (32). The present study clarifies that persons with chronic pain want to be active and recognized as capable, productive partners in care. Accepting support can strengthen their capability, which is also emphasized in person-centred care (32). The capabilities approach focuses on human development and social justice, recognizing that people's capabilities are shaped and formed by environmental and social circumstances (33). A systematic review investigating empirical evidence underpinning the conceptualizations of person-centred care for serious illness found that person-centred care empowers patients *and* their families by providing information and including them in all decision-making actions on their daily lives and care (34).

Methodological considerations

This study has several limitations and strengths. One way to determine trustworthiness in qualitative research is through credibility, dependability, transferability, and conformability (35). Lindseth and Norberg state that phenomenological hermeneutics seeks not to enclose the whole truth but to present meanings of a lived phenomenon vis-à-vis interpreting the narrative text (23). Discussions between the authors ensured the interpretations were plausible while not being the only possibility and that dependability was strengthened. Using the hermeneutic spiral, pending between understanding and explanation by looking at the parts and the whole, helps ensure the credibility of the findings. The study's confirmability was considered because the pre-understanding was noted and used in the comprehensive analysis. Qualitative samples should be large enough to better understand the studied phenomenon but small enough not to hinder qualitative analysis (36). In

For peer review only

phenomenology saturation is not used to determine the number of participants (37); fewer are needed if the data are rich (38). The research group decided that the collected data sufficed to answer the research question and was not too extensive to capture the meaning of the phenomenon. Most participants in the present study were well-educated, female, and born in Sweden, which could affect the generalizability of the findings. Because all participants had lived with pain for many years, they had broad experiences of support and provided rich data, enhancing the study's transferability. However, Bruce et al. described the chronic "pain journey to acceptance" and that different coping mechanisms are useful depending on where the person is in the journey (39), which might also translate to support. Because all participants had lived with pain for many years, they might have gotten further in their journey and the transferability to persons just starting their journey is unclear. The need to use Zoom™ due to the Covid-19 pandemic might have affected the content of the interviews. Still, video interviews are cost-effective and inclusive (28).

Metaphors were used in the interpretation process. Ricoeur contends that the metaphor enriches the meaning of a phenomenon through the creative tension of similarities and differences, generating a new understanding (40). The metaphors demonstrate how language can extend to its limits and affect how we understand the world.

CONCLUSIONS

For persons with chronic pain, support means balancing between being capable (the MVP) and willing to accept support (passing the ball), which aligns with person-centred care. Policymakers, managers, and clinical professionals should consider our findings when planning and implementing care for persons with chronic pain. Future research should focus

For peer review only

on how the healthcare sector can create support to enable persons with chronic pain to be the most valuable player (MVP) while being able to pass the ball in their social networks and healthcare sector.

CONTRIBUTIONS

VL, SW, IE, and ML planned the study. VL performed the data collection. VL, IE, SW, ML, MS, and VS contributed to the data analysis and interpretation of the findings. VL drafted the manuscript and ML, MS, SW, VS, and IE critically reviewed it and contributed to the final version of the manuscript.

ACKNOWLEDGMENTS

The authors gratefully acknowledge the participants in this study for sharing their narratives. We also appreciate the patient organizations (The Swedish Rheumatism Association, The Swedish Association for Survivors of Accident and Injury, Riksförbundet Ehlers-Danlos Syndrom, and The Swedish National Organization for Young Rheumatics) for facilitating the recruitment.

FUNDING

This work was supported by FORTE grant number [2019-00718] and the University of Gothenburg Centre for Person-Centred Care (GPCC), grant number N/A.

For peer review only

COMPETING INTEREST

None declared.

ETHICS APPROVAL

Ethical approval for this study was granted by the Swedish Ethical Review Authority (Reg nr 2020-02491). The study was conducted according to the ethical principles outlined by the Declaration of Helsinki (29).

DATA AVAILABILITY STATEMENT

No additional data are available.

CREATIVE COMMONS LICENCE, CC-BY

For the purpose of Open Access, the authors have applied a CC BY public copyright licence to any Author Accepted Manuscript (AAM) version arising from this submission.

REFERENCES

1. International Association for the Study of Pain I. IASP Revises Its Definition of Pain for the First Time Since 1979 2020 [Available from: https://www.iasp-pain.org/wp-content/uploads/2022/04/revised-definition-flysheet_R2.pdf.

For peer review only

2. Treede RD, Rief W, Barke A, Aziz Q, Bennett MI, Benoliel R, et al. Chronic pain as a symptom or a disease: the IASP Classification of Chronic Pain for the International Classification of Diseases (ICD-11). Pain. 2019;160(1):19-27.
3. Dahlhamer J, Lucas J, Zelaya C, Nahin R, Mackey S, DeBar L, et al. Prevalence of Chronic Pain and High-Impact Chronic Pain Among Adults - United States, 2016. MMWR Morbidity and mortality weekly report. 2018;67(36):1001-6.
4. Foley HE, Knight JC, Ploughman M, Asghari S, Audas R. Association of chronic pain with comorbidities and health care utilization: a retrospective cohort study using health administrative data. Pain. 2021;162(11):2737-49.
5. O'Brien T, Breivik H. The impact of chronic pain-European patients' perspective over 12 months. Scandinavian journal of pain. 2012;3(1):23-9.
6. Fredheim OM, Kaasa S, Fayers P, Saltnes T, Jordhøy M, Borchgrevink PC. Chronic non-malignant pain patients report as poor health-related quality of life as palliative cancer patients. Acta anaesthesiologica Scandinavica. 2008;52(1):143-8.
7. Lundin Å, Ekman I, Wallström S, Andréll P, Lundberg M. Suffering out of sight but not out of mind - interpreting experiences of sick leave due to chronic pain in a community setting: a qualitative study. BMJ open. 2023;13(4):e066617.
8. Breivik H, Collett B, Ventafridda V, Cohen R, Gallacher D. Survey of chronic pain in Europe: prevalence, impact on daily life, and treatment. European journal of pain (London, England). 2006;10(4):287-333.
9. Häuser W, Wolfe F, Henningsen P, Schmutzer G, Brähler E, Hinz A. Untying chronic pain: prevalence and societal burden of chronic pain stages in the general population - a cross-sectional survey. BMC public health. 2014;14:352.
10. de Sola H, Salazar A, Dueñas M, Ojeda B, Failde I. Nationwide cross-sectional study of the impact of chronic pain on an individual's employment: relationship with the family and the social support. BMJ open. 2016;6(12):e012246.
11. Gustavsson A, Bjorkman J, Ljungcrantz C, Rhodin A, Rivano-Fischer M, Sjolund KF, et al. Socio-economic burden of patients with a diagnosis related to chronic pain--register data of 840,000 Swedish patients. European journal of pain (London, England). 2012;16(2):289-99.
12. Devan H, Hale L, Hempel D, Saipe B, Perry MA. What Works and Does Not Work in a Self-Management Intervention for People With Chronic Pain? Qualitative Systematic Review and Meta-Synthesis. Physical therapy. 2018;98(5):381-97.
13. Bair MJ, Matthias MS, Nyland KA, Huffman MA, Stubbs DL, Kroenke K, et al. Barriers and facilitators to chronic pain self-management: a qualitative study of primary care patients with comorbid musculoskeletal pain and depression. Pain medicine (Malden, Mass). 2009;10(7):1280-90.
14. Holtrop JS, Fisher M, Martinez DE, Simpson M, Awadallah NS, Nease DE, Jr., et al. What Works for Managing Chronic Pain: An Appreciative Inquiry Qualitative Analysis. Journal of primary care & community health. 2019;10:2150132719885286.
15. Franklin ZC, Smith NC, Fowler NE. A qualitative investigation of factors that matter to individuals in the pain management process. Disability and rehabilitation. 2016;38(19):1934-42.
16. Pester BD, Tankha H, Caño A, Tong S, Grekin E, Bruinsma J, et al. Facing Pain Together: A Randomized Controlled Trial of the Effects of Facebook Support Groups on Adults With Chronic Pain. The journal of pain. 2022;23(12):2121-34.
17. Matthias MS, McGuire AB, Kukla M, Daggy J, Myers LJ, Bair MJ. A brief peer support intervention for veterans with chronic musculoskeletal pain: a pilot study of feasibility and effectiveness. Pain medicine (Malden, Mass). 2015;16(1):81-7.
18. van Lankveld W, van Helmond T, Näring G, de Rooij DJ, van den Hoogen F. Partner participation in cognitive-behavioral self-management group treatment for patients with rheumatoid arthritis. The Journal of rheumatology. 2004;31(9):1738-45.
19. Riemsma RP, Taal E, Rasker JJ. Group education for patients with rheumatoid arthritis and their partners. Arthritis and rheumatism. 2003;49(4):556-66.

For peer review only

20. Gatchel RJ, Peng YB, Peters ML, Fuchs PN, Turk DC. The biopsychosocial approach to chronic pain: scientific advances and future directions. Psychological bulletin. 2007;133(4):581-624.
21. Cohen SP, Vase L, Hooten WM. Chronic pain: an update on burden, best practices, and new advances. Lancet (London, England). 2021;397(10289):2082-97.
22. Hadi MA, Alldred DP, Briggs M, Marczewski K, Closs SJ. 'Treated as a number, not treated as a person': a qualitative exploration of the perceived barriers to effective pain management of patients with chronic pain. BMJ open. 2017;7(6):e016454.
23. Lindseth A, Norberg A. A phenomenological hermeneutical method for researching lived experience. Scandinavian journal of caring sciences. 2004;18(2):145-53.
24. O'Brien BC, Harris IB, Beckman TJ, Reed DA, Cook DA. Standards for reporting qualitative research: a synthesis of recommendations. Academic medicine : journal of the Association of American Medical Colleges. 2014;89(9):1245-51.
25. Perrot S, Cohen M, Barke A, Korwisi B, Rief W, Treede RD. The IASP classification of chronic pain for ICD-11: chronic secondary musculoskeletal pain. Pain. 2019;160(1):77-82.
26. Palinkas LA, Horwitz SM, Green CA, Wisdom JP, Duan N, Hoagwood K. Purposeful Sampling for Qualitative Data Collection and Analysis in Mixed Method Implementation Research. Administration and policy in mental health. 2015;42(5):533-44.
27. Brinkmann S, Kvale S. InterViews - Learning the Craft of Qualitative Reserach Interviewing. 3rd ed. Los Angeles: SAGE Publications, Inc.; 2015.
28. Saarijärvi M, Bratt EL. When face-to-face interviews are not possible: tips and tricks for video, telephone, online chat, and email interviews in qualitative research. European journal of cardiovascular nursing : journal of the Working Group on Cardiovascular Nursing of the European Society of Cardiology. 2021;20(4):392-6.
29. World Medical Association Declaration of Helsinki: ethical principles for medical research involving human subjects. Jama. 2013;310(20):2191-4.
30. Wieland LS, Manheimer E, Berman BM. Development and classification of an operational definition of complementary and alternative medicine for the Cochrane collaboration. Alternative therapies in health and medicine. 2011;17(2):50-9.
31. Hsu C, Sherman KJ, Eaves ER, Turner JA, Cherkin DC, Cromp D, et al. New perspectives on patient expectations of treatment outcomes: results from qualitative interviews with patients seeking complementary and alternative medicine treatments for chronic low back pain. BMC complementary and alternative medicine. 2014;14:276.
32. Ekman I. Practising the ethics of person-centred care balancing ethical conviction and moral obligations. Nursing philosophy : an international journal for healthcare professionals. 2022;23(3):e12382.
33. Entwistle VA, Watt IS. Treating patients as persons: a capabilities approach to support delivery of person-centered care. The American journal of bioethics : AJOB. 2013;13(8):29-39.
34. Giusti A, Nkhoma K, Petrus R, Petersen I, Gwyther L, Farrant L, et al. The empirical evidence underpinning the concept and practice of person-centred care for serious illness: a systematic review. BMJ global health. 2020;5(12).
35. Lincoln Y, Guba E. Naturalistic Inquiry. Newbury Park, CA: SAGE Publications, Inc.; 1985.
36. Sandelowski M. Sample size in qualitative research. Research in nursing & health. 1995;18(2):179-83.
37. van Manen M, Higgins I, van der Riet P. A conversation with Max van Manen on phenomenology in its original sense. Nursing & health sciences. 2016;18(1):4-7.
38. Morse JM. Determining Sample Size. Qualitative health research. 2000;10(1):3-5.
39. Bruce M, Lopatina E, Hodge J, Moffat K, Khan S, Pyle P, et al. Understanding the chronic pain journey and coping strategies that patients use to manage their chronic pain: a qualitative, patient-led, Canadian study. BMJ open. 2023;13(7):e072048.
40. Ricoeur P. The rule of metaphor. Abingdon: Routledge Press; 2003.

For peer review only

For peer review only

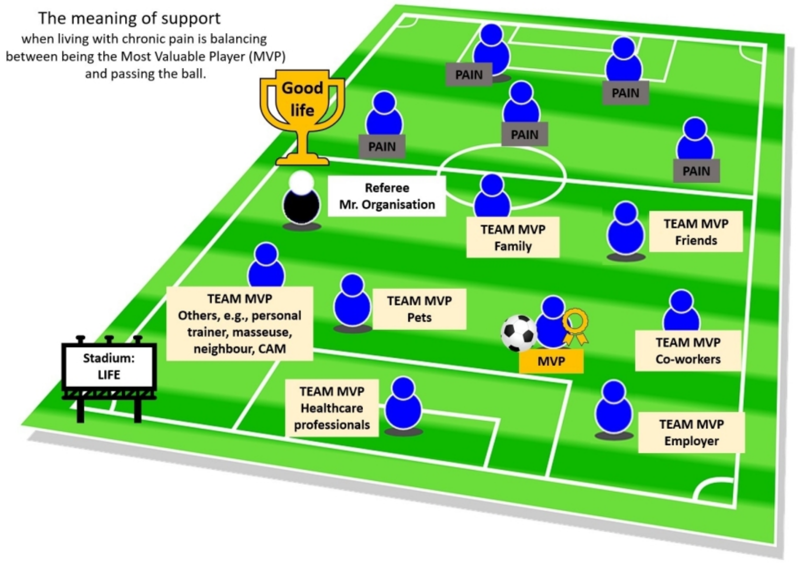

Figure 1. The match against pain. Most Valuable Player/MVP (person with chronic pain) with the ball (pain management), the team (examples of teammates), the opponent (pain), the referee (representing fair conditions), the stadium (life), and the trophy (symbolising the aspiration for a good life despite living with chronic pain).

162x91mm (300 x 300 DPI)

Pre-understanding

I believe persons with chronic pain often feel as if they have been treated poorly in encounters with healthcare professionals. I think they perceive that their pain is not taken seriously and that they are not being believed. I also think they perceive they are not being given the required help and struggle to get help. I believe they mostly have been in contact with medical doctors.

I believe they experience that their social network is not being invited to participate in their care. I think they feel support from their social network without including the network within their care. I do not know how they perceive support, but I can imagine that the employer, family, and friends are significant and their relationships are often impacted by pain. People with chronic pain want to involve their social network if healthcare professionals ask them.

For peer review only

Interview guide

The aim is to elucidate the meaning of lived experiences of support from the social network and healthcare sector in persons with chronic pain.

Research questions

How do persons with chronic pain experience support from the healthcare sector?

How do persons with chronic pain experience support from their social network outside the healthcare sector?

How do persons with chronic pain perceive involving their social network in their care and rehabilitation?

Support within the healthcare sector

Do you want to tell me when you first contacted the healthcare sector regarding your pain?

How have you perceived the help you've gotten from the healthcare sector since then?

- What did you feel when you did/did not receive that help?
- How has the help you've received from the healthcare sector made a difference to you?

What kind of help do you feel you would have liked to have but have not gotten from the healthcare sector?

- Are there any symptoms you would have liked to receive more help with?
- How did you feel when you received/did not receive that help?

For peer review only

How would you like the healthcare sector to help you?

Support from the social network

Which persons outside of the healthcare sector have been important to you regarding your pain?

- How has contact with those people helped you?
- What kind of difference has that contact made for you?

Are there others outside the healthcare sector who have been important to you regarding your pain?

- How has contact with these people helped you?
- What difference has the contact with these persons made for you?

Are there any persons outside the healthcare sector you would have liked to receive more support from?

- Do you want to tell me more about this?
- What did you feel when you did not receive that support?

Involvement of the social network in care

How have persons from outside the healthcare sector been involved in your care? (for instance, next of kin).

- In which parts of the care have they been involved?
- How did it come about that they got involved?

For peer review only

- Who initiated the involvement?

How has it worked to have them involved in your care?

- How have you experienced it?

How do you feel about involving persons outside of the healthcare sector in your care?

- In which situations would you like to involve persons from outside the healthcare sector in your care?

What would facilitate involving other persons in your care?

How do you perceive the healthcare sector's attitude towards involving other persons?

Concluding

Is there anything that we've discussed that you would like to talk more about or something we haven't addressed that you would like to add?

For peer review only

Standards for Reporting Qualitative Research (SRQR)*

http://www.equator-network.org/reporting-guidelines/srqr/

Title and abstract

	Page/line no(s).
**Title** - Concise description of the nature and topic of the study Identifying the study as qualitative or indicating the approach (e.g., ethnography, grounded theory) or data collection methods (e.g., interview, focus group) is recommended	p1 / 1-3
**Abstract** - Summary of key elements of the study using the abstract format of the intended publication; typically includes background, purpose, methods, results, and conclusions	p3 / 1-16

Introduction

**Problem formulation** - Description and significance of the problem/phenomenon studied; review of relevant theory and empirical work; problem statement	p5 / 1-23<br>p6 / 1-19
**Purpose or research question** - Purpose of the study and specific objectives or questions	p6 / 20-21

Methods

**Qualitative approach and research paradigm** - Qualitative approach (e.g., ethnography, grounded theory, case study, phenomenology, narrative research) and guiding theory if appropriate; identifying the research paradigm (e.g., postpositivist, constructivist/ interpretivist) is also recommended; rationale**	p7 / 2-5
**Researcher characteristics and reflexivity** - Researchers' characteristics that may influence the research, including personal attributes, qualifications/experience, relationship with participants, assumptions, and/or presuppositions; potential or actual interaction between researchers' characteristics and the research questions, approach, methods, results, and/or transferability	p8 / 7-12
**Context** - Setting/site and salient contextual factors; rationale**	p7 / 9-11
**Sampling strategy** - How and why research participants, documents, or events were selected; criteria for deciding when no further sampling was necessary (e.g., sampling saturation); rationale**	p7/ 11-23<br>p8 / 1-6
**Ethical issues pertaining to human subjects** - Documentation of approval by an appropriate ethics review board and participant consent, or explanation for lack thereof; other confidentiality and data security issues	p9 /9-16
**Data collection methods** - Types of data collected; details of data collection procedures including (as appropriate) start and stop dates of data collection and analysis, iterative process, triangulation of sources/methods, and modification of procedures in response to evolving study findings; rationale**	p8 / 21-23<br>p9 / 1-3

For peer review only - http://bmjopen.bmj.com/site/about/guidelines.xhtml

**Data collection instruments and technologies** - Description of instruments (e.g., interview guides, questionnaires) and devices (e.g., audio recorders) used for data collection; if/how the instrument(s) changed over the course of the study	p8 / 14-23
**Units of study** - Number and relevant characteristics of participants, documents, or events included in the study; level of participation (could be reported in results)	p11 / 8-12<br>p12 / 1<br>p13 / 1
**Data processing** - Methods for processing data prior to and during analysis, including transcription, data entry, data management and security, verification of data integrity, data coding, and anonymization/de-identification of excerpts	p8 / 23<br>p9 / 14-16<br>p10 / 5-6
**Data analysis** - Process by which inferences, themes, etc., were identified and developed, including the researchers involved in data analysis; usually references a specific paradigm or approach; rationale**	p7 / 4-5<br>p9 / 18-22<br>p10 / 1-16<br>p11 / 1-6
**Techniques to enhance trustworthiness** - Techniques to enhance trustworthiness and credibility of data analysis (e.g., member checking, audit trail, triangulation); rationale**	p8 / 8-12<br>p9 / 22<br>p10 / 1-2 & 8-13<br>P11 / 2-6

Results/findings

**Synthesis and interpretation** - Main findings (e.g., interpretations, inferences, and themes); might include development of a theory or model, or integration with prior research or theory	p13 / 6-15<br>p14 / 1-21<br>p15 / 1-18<br>p16 / 1-22<br>p17 / 1-23<br>p18 / 18-23<br>p19 / 1-22<br>p20 / 1-23<br>p21 / 1-16
**Links to empirical data** - Evidence (e.g., quotes, field notes, text excerpts, photographs) to substantiate analytic findings	p16 / 4-6<br>p17 / 8-11<br>p18 / 8-11<br>p19 / 3-6 & 21-22<br>p20 / 1-2

Discussion

**Integration with prior work, implications, transferability, and contribution(s) to the field -** Short summary of main findings; explanation of how findings and conclusions connect to, support, elaborate on, or challenge conclusions of earlier scholarship; discussion of scope of application/generalizability; identification of unique contribution(s) to scholarship in a discipline or field	p21 / 20-22<br>p22 / 1-24<br>p23 / 1-10
**Limitations** - Trustworthiness and limitations of findings	p23 / 12-23<br>p24 /1-17

Other

**Conflicts of interest** - Potential sources of influence or perceived influence on study conduct and conclusions; how these were managed	p26 / 1-2

For peer review only - http://bmjopen.bmj.com/site/about/guidelines.xhtml

For peer review only

**Funding** - Sources of funding and other support; role of funders in data collection, interpretation, and reporting	p25 / 18-20

*The authors created the SRQR by searching the literature to identify guidelines, reporting standards, and critical appraisal criteria for qualitative research; reviewing the reference lists of retrieved sources; and contacting experts to gain feedback. The SRQR aims to improve the transparency of all aspects of qualitative research by providing clear standards for reporting qualitative research.

**The rationale should briefly discuss the justification for choosing that theory, approach, method, or technique rather than other options available, the assumptions and limitations implicit in those choices, and how those choices influence study conclusions and transferability. As appropriate, the rationale for several items might be discussed together.

Reference:
O'Brien BC, Harris IB, Beckman TJ, Reed DA, Cook DA. **Standards for reporting qualitative research: a synthesis of recommendations.** *Academic Medicine*, Vol. 89, No. 9 / Sept 2014 DOI: 10.1097/ACM.0000000000000388

For peer review only

Balancing between being the Most Valuable Player (MVP) and passing the ball: A qualitative study of support when living with chronic pain in Sweden

Journal:	*BMJ Open*
Manuscript ID	bmjopen-2023-079229.R1
Article Type:	Original research
Date Submitted by the Author:	10-Nov-2023
Complete List of Authors:	Lilja, Veronica; University of Gothenburg Institute of Health and Care Sciences, Sahlgrenska Academy; University of Gothenburg Centre for Person-Centred Care, Sahlgrenska Academy<br>Wallström, Sara; University of Gothenburg Institute of Health and Care Sciences, Sahlgrenska Academy; University of Gothenburg Centre for Person-Centred Care, Sahlgrenska Academy<br>Saarijärvi, Markus; Uppsala University Department of Women's and Children's Health; University of Gothenburg Centre for Person-Centred Care, Sahlgrenska Academy<br>Lundberg, Mari; Sophiahemmet University, Department of Health Promoting Science; University of Gothenburg Centre for Person-Centred Care, Sahlgrenska Academy<br>Segertoft, Vivi-Anne; Personskadeförbundet,<br>Ekman, Inger; University of Gothenburg Institute of Health and Care Sciences; University of Gothenburg Centre for Person-Centred Care
**Primary Subject Heading**:	Qualitative research
Secondary Subject Heading:	Nursing
Keywords:	Chronic Pain, Social Support, QUALITATIVE RESEARCH, PAIN MANAGEMENT

SCHOLARONE™ Manuscripts

I, the Submitting Author has the right to grant and does grant on behalf of all authors of the Work (as defined in the below author licence), an exclusive licence and/or a non-exclusive licence for contributions from authors who are: i) UK Crown employees; ii) where BMJ has agreed a CC-BY licence shall apply, and/or iii) in accordance with the terms applicable for US Federal Government officers or employees acting as part of their official duties; on a worldwide, perpetual, irrevocable, royalty-free basis to BMJ Publishing Group Ltd ("BMJ") its licensees and where the relevant Journal is co-owned by BMJ to the co-owners of the Journal, to publish the Work in this journal and any other BMJ products and to exploit all rights, as set out in our licence.

The Submitting Author accepts and understands that any supply made under these terms is made by BMJ to the Submitting Author unless you are acting as an employee on behalf of your employer or a postgraduate student of an affiliated institution which is paying any applicable article publishing charge ("APC") for Open Access articles. Where the Submitting Author wishes to make the Work available on an Open Access basis (and intends to pay the relevant APC), the terms of reuse of such Open Access shall be governed by a Creative Commons licence – details of these licences and which Creative Commons licence will apply to this Work are set out in our licence referred to above.

Other than as permitted in any relevant BMJ Author's Self Archiving Policies, I confirm this Work has not been accepted for publication elsewhere, is not being considered for publication elsewhere and does not duplicate material already published. I confirm all authors consent to publication of this Work and authorise the granting of this licence.

For peer review only

Balancing between being the Most Valuable Player (MVP) and passing the ball: A qualitative study of support when living with chronic pain in Sweden

Veronica Lilja, corresponding author (Orcid ID 0000-0002-5531-6358)

E-mail: veronica.lilja@gu.se

- Institute of Health and Care Sciences, Sahlgrenska Academy, University of Gothenburg, Gothenburg, Sweden
- University of Gothenburg Centre for Person-Centred Care (GPCC), Sahlgrenska Academy, University of Gothenburg, Gothenburg, Sweden

Sara Wallström: Orcid ID:0000-0001-7579-4974

- Institute of Health and Care Sciences, Sahlgrenska Academy, University of Gothenburg, Gothenburg, Sweden
- University of Gothenburg Centre for Person-Centred Care (GPCC), Sahlgrenska Academy, University of Gothenburg, Gothenburg, Sweden

Markus Saarijärvi: Orcid ID: 0000-0002-7053-6930

- Department of Women's and Children's Health, Uppsala University, Uppsala, Sweden
- University of Gothenburg Centre for Person-Centred Care (GPCC), Sahlgrenska Academy, University of Gothenburg, Gothenburg, Sweden

For peer review only

Mari Lundberg: Orcid ID 0000-0001-7123-0362

- Department of Health Promoting Science, Sophiahemmet University, Stockholm, Sweden
- University of Gothenburg Centre for Person-Centred Care (GPCC), Sahlgrenska Academy, University of Gothenburg, Gothenburg, Sweden

Vivi-Anne Segertoft: Orcid ID 0009-0009-7916-668X

- The Swedish Association for Survivors of Accident and Injury, Uddevalla, Sweden

Inger Ekman: Orcid ID: 0000-0002-5559-5203

- Institute of Health and Care Sciences, Sahlgrenska Academy, University of Gothenburg, Gothenburg, Sweden
- University of Gothenburg Centre for Person-Centred Care (GPCC), Sahlgrenska Academy, University of Gothenburg, Gothenburg, Sweden

For peer review only

ABSTRACT

Objective: This study aimed to elucidate the meaning of lived experiences of support from social networks and the healthcare sector in persons with chronic pain.

Design: A qualitative, phenomenological hermeneutic method was used to analyse interview data.

Setting: Participants were recruited from patient organisations in Sweden.

Participants: Ten (seven women, two men and one non-binary) individuals with chronic musculoskeletal pain were included.

Findings: The meaning of lived experiences of support in persons with chronic pain involves balancing between being the most valuable player (MVP) and passing the ball, meaning balancing between being a capable person and accepting support to be that capable person.

Conclusion: For participants who lived with chronic pain, support means balancing between being capable (the MVP) and willing to accept support (passing the ball), which aligns with the concept of person-centred care. Our findings may be useful for policymakers, managers, and clinical professionals when planning and performing care for persons with chronic pain. Future research should focus on how the healthcare sector can create support to enable persons with chronic pain to be the most valuable players (MVP) while being able to pass the ball to their social networks and the healthcare sector.

Keywords: chronic pain, social support, qualitative research, pain management

STRENGTHS AND LIMITATIONS

- Chronic pain affects many people worldwide and understanding the meaning of the healthcare sector and social network support is vital to provide tailored assistance and practical solutions.

- Further insights were achieved with a patient representative actively involved in the analysis and manuscript preparation.

For peer review only

- A diversity of basic demographic indicators (age, geographic location, and occupational status) is a strength of the study.

- A limitation is that most participants had post-high school education, were female (all eligible participants with other genders were included) and were born in Sweden.

For peer review only

INTRODUCTION

Pain is defined as "an unpleasant sensory and emotional experience associated with, or resembling that associated with, actual or potential tissue damage" (1). Chronic pain persists or recurs for over 3 months (2) and is classified as a disease on its own, not just a symptom. The prevalence of chronic pain differs between studies, contexts, and types of measurement. In a large European study comprising 16 countries the prevalence of chronic pain was estimated to be 19% (3). A US study showed a prevalence rate of 20.4% (4).

Persons with chronic pain often have comorbidities (e.g., depression, anxiety, cardiovascular disease, and cancer) (5), side effects from medication (6), and poor health-related quality of life (7). Chronic pain can adversely affect sleep, daily activities, relationships, and the ability to work (6). Pain is often perceived as invisible to others, which can contribute to feeling unjustly treated in society (8). From a societal and health economic perspective, chronic pain presents challenges because it is a common reason for sick leave (3, 9, 10) and healthcare-seeking behaviour (5, 6, 9). In Sweden, the cost (indirect and direct) of chronic pain-related diagnoses was estimated at 32 billion euros per year in 2012, of which 59% were due to sick leave and early retirement (11).

A meta-synthesis showed that support from family and friends is important in pain management (12). Social support can include sharing advice, expressions of empathy, and contributing to positive feelings (13, 14). In contrast, lacking support can lead to feelings of loneliness, and not being needed (15). Peer support interventions have been shown to decrease pain severity and interference (16). However, there is conflicting evidence of the positive effects of support. A peer support intervention for veterans with musculoskeletal pain found no statistically significant impact on pain (17). Studies investigating spouses'

For peer review only

participation in educational interventions suggest no additional benefits of including a partner (18) and that participating with a partner could make participants more prone to fatigue and lower self-efficacy compared to not participating with a partner (19).

Collaborative relationships with health care professionals constitute support that facilitates self-management of pain (12). The biopsychosocial model and the multimodal approach have been shown to improve pain management (20, 21). However, it has also been reported that persons with chronic pain feel that healthcare professionals rarely take their condition seriously and that they desire better support from health professionals (3, 15, 22).

Due to conflicting evidence and the complexity of support for persons with chronic pain, there is a need to understand the meaning of support, both within and outside the healthcare system. A deeper understanding of the phenomenon would facilitate the comprehension of the need for support and could aid in bringing clarity on what kind of support persons with chronic pain want and need.

Therefore, this study aims to elucidate the meaning of lived experience of support from social networks and the healthcare sector in persons with chronic pain.

METHODS

Design

The present study applied a qualitative method with a phenomenological hermeneutic approach inspired by Lindseth and Norberg (23). Phenomenological hermeneutics is suitable

For peer review only

for interpreting the essential meaning of a lived phenomenon through text narratives (23). This study follows the Standards for Reporting Qualitative Research guidelines (24).

Participants and setting

Participants were recruited from four Swedish patient organisations by a Facebook post or an e-mail sent from the organisations. These organisations are well established, with members having different diagnoses and many having chronic pain in common. Persons willing to participate in the study contacted the first author (VL) by e-mail. Inclusion criteria were ≥18 years of age, living in Sweden and having chronic musculoskeletal pain (defined as “chronic pain arising from musculoskeletal structures” (25)). Persons who primarily seemed to struggle with other conditions and congenital diseases, such as concurrent cancer diagnosis, were excluded. Participants who mainly wanted to share their narratives about musculoskeletal pain but had congenital diseases, undergone cancer treatment or had another pain-related diagnosis were not excluded.

Some 177 persons (1 non-binary, 4 men, 172 women) expressed interest in participating. A purposive sampling strategy was employed to include participants from different parts of Sweden, regardless of treatment or current contact with healthcare. Five participants mentioned receiving support from the healthcare sector and social networks by starting to share their narratives in the e-mail expressing interest in participating. They were purposefully selected as they were willing to share their vast experience of the phenomenon under study, allowing the collection of rich data (26). Maximum variation sampling allowed the discovery of common meanings across demographic differences (26). Therefore, a diversity of experiences of support, age, geographic location, sick leave rate,

For peer review only

and background diagnosis was sought even though most potential participants were women. Eight participants were initially included, and their narratives were deemed sufficient to answer the study's research question. Another two participants were interviewed to achieve greater variation in education level. None of the participants declined to participate. The material was considered rich enough to find meanings of support. After discussions in the research group, inclusion was halted at 10 participants.

The author who conducted the interviews (VL) has a nursing and public health background. Before the study, the interviewer's pre-understanding was written (see supplemental file 1) and reflected upon in the analysis. VL was a novice in phenomenological hermeneutics; however, the research group's extensive experience complemented her lack of practice in this field. A patient representative was also part of the research group and contributed with experience of living with chronic pain.

Data collection

A semi-structured interview guide (supplemental file 2), derived from Brinkmann and Kvale (27), was developed by VL with input from SW, ML and IE. The guide included three domains of support: the healthcare sector, social networks, and how support from social networks could be integrated into care. The three domains were chosen based on their previously described importance (3, 12-16, 19-22). The interview guide contained open-ended questions with suggestions for additional probing questions. It was piloted in the first two interviews and revised by changing from the question "Which persons outside of the healthcare sector have you gotten support/help from to "Which persons outside of the healthcare sector have been important to you regarding your pain?" The final version of the

For peer review only

question better facilitated narratives about social networks. The narratives from the two pilot interviews were deemed relevant, as they answered the research question and were thus included in the data analysis. Seven interviews were conducted digitally through Zoom™, one by phone, and two face-to-face between February 2021 and August 2022. The interviews were recorded and transcribed verbatim by VL. Participants chose the interview date, place, and format. Video and telephone interviews are trustworthy alternatives to face-to-face interviews in qualitative research (28).

Patient and public involvement

Four patient organisations contributed to recruiting study participants and will contribute to disseminating the findings. One of the co-authors (VS) is a patient representative. VS actively participated in data analysis and manuscript preparation.

Ethical considerations

Ethical approval for this study was granted by the Swedish Ethical Review Authority (Reg nr 2020-02491). In addition, the study was conducted according to the ethical principles outlined in the Declaration of Helsinki (29). All participants received written and verbal information about the study. Written and verbal informed consent was obtained from all participants and all data were handled confidentially. Participants' identities were removed during transcription. Audio recordings, transcripts, and the keycode were safely stored in different locations to protect the participants' identity and personal information.

For peer review only

Data analysis

Data were analysed with phenomenological hermeneutics. The method, influenced by Ricoeur's theory of interpretation and developed by Lindseth and Norberg (23), involves three intertwined phases: *naïve understanding*, *structural analysis,* and *comprehensive understanding*. Through the hermeneutic spiral, the phases are constantly overlapping, revisited and compared to each other to move between explanation and understanding by interpretation of the whole and the parts (30).

Naïve understanding

Each interview was read several times. VL formulated a naïve understanding for each interview before constructing a merged naïve understanding of all interviews.

Structural analyses

The structural analyses were performed with the software Nvivo version 12 by VL with input from the other authors. The text of each interview was divided into meaning units and condensed. All text was considered, but only text associated with the study's aim was included in the structural analyses. The condensed meaning units were continuously compared to the naïve understanding. The interviews were read through again, and the naïve understanding was revised and compared to the structural analyses. This process was repeated several times. Eventually, tentative themes and sub-themes were formulated and compared to the condensed meaning units and the naïve understanding. VL and IE

For peer review only

continuously discussed and reformulated the tentative findings before consulting the other authors.

Table 1. Examples of structural analysis.

Text	Condensed meaning units	Sub-theme	Theme	Main theme
The appreciation of myself was destroyed already at the beginning of my sickness … and the value… my own value. Because they did not see me as competent. They saw only my illness. So, this has been awfully hard. And there is still frustration in not getting this kind of recognition.	Self-worth	Being a valuable player and not only being the injured one.	Being the MVP	Balancing between being the MVP and passing the ball in a match against pain
I was so incredibly fortunate; he is a great guy, a great doctor and, and… he supports me in the next step.	Someone fights with you	Being able to pass the ball when you have to	Passing the ball	Balancing between being the MVP and passing the ball in a match against pain

Interpretation of the whole

In interpreting the whole VL and IE compared the pre-understanding, the naïve understanding, and the structural analyses several times to identify inconsistencies. The

For peer review only

analysis was revised until all parts were consistent. The underlying meaning in the data was reflected on and compared to the existing literature, such as previous studies, the work of the philosopher Ricoeur, and the underpinnings of person-centred care, yielding a new understanding. ML read all the interviews and the findings to ensure the interpretations were reasonable before giving feedback on the naïve understanding, structural analyses, and interpretation of the whole. The understanding of the meaning of the findings was discussed among all authors several times to connect their perspectives, knowledge, and understandings. The interpreted metaphor was developed through discussions among all authors based on the link to the naïve understanding and structural analyses. When consensus on the meaning of the findings and the metaphor was reached, the interpretation of the whole was formulated.

FINDINGS

Ten participants were included in the study (two men, one non-binary and seven women). All eligible participants of other genders than female were included. The demographic characteristics of participants are described in Table 2. The interviews lasted between 39 and 101 minutes (mean 77 minutes).

Table 2. Participant characteristics.

Characteristics	
**Age**	
Mean (range)	48 years (24-70)

For peer review only

**Sex**	
Female	7
Male	2
Non-binary	1
**Place of birth**	
Born in Sweden	9
Not born in Sweden	1
**Diagnosis**	
Fibromyalgia	1
Post-polio syndrome	2
Spinal injury	1
Ehlers-Danlos Syndrome (hEDS and HSD)	4
Fibromyalgia and Ehlers-Danlos Syndrome	2
**Duration of pain**	
Mean (range)	32.6 years (14-66)
**Education level**	
High school degree	3
University degree	7
**Occupational status**	
Working full-time	5

For peer review only

Working part-time due to sick leave	3
Unemployed without financial support	1
On disability pension	1
**Relationship status**	
Partner	7
Single	3

Findings from the analyses are presented in the following order: the naïve understanding, the structural analyses, and the interpretation of the whole.

Naïve understanding

The naïve understanding of the meaning of support is that it reinforces participants' ability to manage their pain and everyday life. Participants seek to address their pain and life situation independently but need support to achieve these goals. They feel lonely in and diminished by the healthcare sector, often seen as unavailable or hostile. Support from the right healthcare professional, someone who listens and will go the extra mile to establish a diagnosis and provide help, makes a big difference in participants' perception of their own capability. Experiences of support from outside the healthcare sector vary considerably. Although participants are eager to manage independently, social networks that believe in them, show compassion, and fight together with them are essential.

For peer review only

Structural analyses

The main theme, themes, and sub-themes are described below and in Table 3. The metaphor of a football match is used throughout the designation of the main theme, themes and sub-themes to elucidate the meaning of support. The metaphor is related to participants' narratives and elaborated on under each heading.

Balancing between being the Most Valuable Player (MVP) and passing the ball in the match against pain

Chronic pain can be a constant battle, and just like a football match, it can vary in intensity. In this match pain is the opponent, the person with chronic pain is the MVP (Most Valuable Player), and the teammates are individuals within the MVP's social networks and healthcare sector. The social networks could include family, partners, friends, employers, colleagues, peers with chronic pain, personal trainers, personal coaches, persons performing complementary therapies, neighbours, and pets. The ball (designated as pain management) in this football match is passed around to members of the MVP's team to win the match against pain. The attempt to win the match does not mean being pain-free but living the life the person with chronic pain wants to live despite the pain. The meaning of lived experiences of support is the constant balancing act between managing alone (being the MVP) and accepting help from others (passing the ball), which is further explained through the themes and sub-themes.

For peer review only

Table 3. Overview of the main theme, themes, and sub-themes.

Main theme: Balancing between being The Most Valuable Player (MVP) and passing the ball in the match against pain	
Theme: Being the MVP	Theme: Passing the ball
Sub-theme: Being a valuable player and not just the injured one	*Sub-theme: Being part of a team*
Sub-theme: Being trusted to dribble	*Sub-theme: Having teammates when you have been tackled*
	Sub-theme: Worrying about being a benchwarmer

For peer review only

Being the MVP

Participants wanted to contribute to society like everyone else and manage independently by taking the lead in their care and daily life. They also desired to be who they were without pain dominating their lives. Contemplating the metaphor, this can be interpreted as they aspired to be the MVP in all aspects of their lives. Being believed and listened to were important aspects of being trusted to dribble the ball, which is essential when seeking to be the MVP.

Being a valuable player and not just the injured one

Participants sought to be recognised as the persons they were, with unique personalities and experiences, which could often be difficult to achieve. When perceived as a product of their pain, they felt excluded and viewed as someone who could not accomplish much.

When the social networks provided support by accepting the pain as part of the participants but still recognising them for who they were and their capabilities, it facilitated their acceptance of themselves and the pain.

"They used to ask me,' Why are you limping?' I said,' I played football last weekend,' and I have never played football. So he [participant's partner] told me, 'Now you're going to stop to tell them that you've played football.' After that, I felt fine. There was no problem. I even have a colleague who says, 'You're limping.' Maybe you should sit down.' I don't have to be perfect all the time."

- Participant 4.

Pets could also contribute to this support, providing unconditional affection and companionship. Participants also wanted health care professionals to recognise them as the persons they were. They felt supported when healthcare professionals aimed at strengthening their resources by not only focusing on their limitations but also on their abilities. Participants felt an enhanced power by accessing self-help devices (e.g., orthoses).

"It is about being an important part of society, to contribute… instead of being the one others should take care of. Self-help devices help to achieve that."

- Participant 10.

The social networks provided support by requesting the participants' help and advice. This support affirmed the participants' view of themselves as unique, capable, meaningful, and contributors rather than just someone with pain. Providing peer support to others with pain within a patient organisation exemplifies how participants contributed.

For peer review only

"I find good support in supporting others. You get a reflection of yourself that way. So, maybe that is my best support, to support others. […] It was perfect to have somebody who needed me."

- Participant 3.

Being recognised as a person rather than someone struggling with pain could be interpreted as being considered a valuable player in football. Valuable football players are still useful to the team when injured, as everyone appreciates their efforts and knows their potential. They are not regarded as "that injured player." They are still valuable, and everyone is eager to see them return to the field.

Being trusted to dribble

To participants a diagnosis was important to have their pain experiences taken seriously, understanding their pain, and being believed, but it was often perceived as a challenging process. A diagnosis meant validating their condition and was also experienced as facilitating being believed, trusted, and understood by the social networks. Being believed, trusted, and understood by others was a support, and it also encouraged that expectations from others did not clash with the participants' abilities. When participants were trusted with tasks they could perform, their view of themselves as capable persons was reinforced.

"I have to put on a mask in front of people and pretend to be happy. But my friend says, "I don't mind that you're low and in pain. You don't need to be happy and energised. We can still have coffee, like getting rid of a 20 kg backpack. He understands."

- Participant 7.

For peer review only

Participants sometimes felt they received better support within complementary therapies compared to traditional care. They felt listened to and perceived as capable. This complimentary support, however, was not always affordable because of the participants' often strained economy.

"My massage therapist sees me. I think that is a need that everyone has, to be seen and listened to."

- Participant 2.

Being believed and listened to when sharing experiences of pain could be viewed as a football player being trusted by other teammates. The teammates show that the player and their abilities are trusted by passing them the ball and encouraging them to dribble, meaning taking responsibility for the next move.

Passing the ball

Living with pain was challenging, and occasionally, participants felt their abilities were inadequate. with their situation. They needed a team to help them regain trust in themselves and fight to improve their situation. In football the MVP must have a team to pass the ball to because they cannot win the game alone.

Being part of a team

Participants often felt alone, struggling with pain. Navigating the healthcare system and the social insurance agency's policies was difficult. Fighting different systems alone evoked feelings of being diminished, vulnerable, and powerless. When they found regular contact

For peer review only

with a health care professional willing to help and possibly involve other professionals, or when their networks helped them fight the system, they did not feel as lonely. Regular contact enhanced the participants' sense of capability. Having support from others, they felt more confident and could accomplish more.

"My personal trainer said, 'I have a plan. Let's focus on this so you can continue fighting.' Thanks to her, I am stronger and I feel like things are moving forward."

- Participant 1.

"I want to take responsibility for my pain and situation myself, but to be able to have someone to ask for help when I can't bear to deal with it. Like a backup, a support that stands on the side but does not overtake the main responsibility. It is very easy to end up in a subordinate position when you have a chronic condition, that you're in the hands of others. I want to be in charge but still have that support system around me as a backup for when I'm worse. When I do not have that, I have to push myself beyond my limits, and my health deteriorates."

- Participant 8.

Reducing loneliness by accepting support from others could be analogous to being part of a football team. The individual is stronger with teammates and has a better chance of beating the other team (i.e., overcoming chronic pain). Being part of a team also means being included and not alone.

For peer review only

Having teammates when you have been tackled

Participants felt hopeless when told by health care professionals there was nothing left to be done after undergoing several medical treatments or when test results failed to reveal the cause of their pain. The hope of feeling capable was restored when they were provided support (e.g., tailored training programmes or self-help devices). When social networks provided support by facilitating daily tasks, tailored work schedules, ideas for pain management, or contacts with trusted health care professionals, participants felt hope that their circumstances would improve, and they could live the desired life.

"I was advised to change my diet. It helped me so much. It somewhat improved my state because I could affect my situation a little bit myself."

- Participant 5.

Regaining hope could mean having a teammate take the ball when the football player has been tackled and cannot dribble on their own (when the pain is unbearable, and they do not know how to move on). By having a teammate take the ball (pain management), the hope remains that the team can control the ball instead of losing it to the opposing team (the pain). There is hope that there is something else to try, even though all options for pain management are exhausted and someone is fighting with you when you feel like your abilities are not enough to fight the pain on your own.

"When I get this lumbago, I believe it will never pass, and I have to live my whole life like this. And then I talk to him [participant's partner], and he reminds me it will pass, and I'll be alright. It is good to be reminded. Otherwise, I'll go into that tunnel, thinking everything will go bad."

For peer review only

- Participant 4.

Worrying about being a benchwarmer

Participants found it challenging to determine what they could share about their situation with their networks (particularly family and friends) without appearing as a burden. Not wanting to strain their networks or cause worry discouraged participants from including them in their care.

"I don't know how much is okay to… share. What is oversharing? And how much can I share so it becomes enough, so they understand, but it does not become too much? It is a balancing act, and I find it difficult to know where the boundaries are. I dare not take that leap to tell them about my situation."

- Participant 6.

Some health care professionals blamed participants for their situation, whereas others went beyond their regular duties to support participants by working during their free time (e.g., lunch break). Such support made a huge difference, as participants needed their help. At the same time, the feeling of being a burden was enhanced because the health care professionals had to sacrifice their spare time to offer that support. Fear of being a burden due to pain could be seen as fear of losing the title of MVP and becoming a benchwarmer, i.e., always on the bench without the opportunity to participate and contribute.

"You are sort of like a ball being kicked around within the system. And even if you try to tell them 'No, this is the field, this is the playing field', it becomes a bit like whatever. […] But I have been so lucky that these people are helping me out of pure, goodhearted will, even

For peer review only

though they do not have time for it. Because their bosses tell them, 'You should only do this or that because these are our resources.' And I mean, they even helped me during lunch and such things. I must be careful when asking them for help because they are so kind-hearted." - Participant 9.

Interpretation of the whole

As the main theme suggests, the meaning of the participants' lived experiences of support from the healthcare sector and social networks is to balance being a capable person and accepting help from others to continue being that capable person. Being capable means being recognized for who you are as a person and your qualities, to contribute, to be trusted and listened to. Accepting support means to not be alone, having someone fighting with you in order to enhance one's capability, but also to worry about being a burden. In football terms, being capable corresponds to being the MVP and accepting support corresponds to passing the ball. Developing the interpreted football metaphor; the MVP and the team compete against pain (see Figure 1).

Many football players dream of being the MVP, whose actions ultimately determine the match's outcome. Our interpretation of the findings is that persons with chronic pain are no different, i.e., they aspire to be important. However, not even the MVP can win a match without team support. They need the team to believe in their capability and support them to reach their full potential.

In accordance with how the MVP takes the lead, we would argue that persons with chronic pain take the lead in their daily lives and care by dribbling the ball (pain management). However, there must be a balance, as no team will win a match through only having the

For peer review only - http://bmjopen.bmj.com/site/about/guidelines.xhtml

MVP dribbling, i.e., it is also necessary to pass the ball. Passing the ball does not mean losing the title as MVP; instead, it means boosting the chances of winning the match through the help of teammates. Living with chronic pain often leads to accepting a life with pain. Aspiring to win the match against pain is not about being pain-free but about living a meaningful life and being capable despite the pain.

When social networks and healthcare professionals do not listen to persons with chronic pain, believe them, see their capabilities, or claim they can no longer be helped, they go to the opposing team. The person with chronic pain might also end up on the opposing team by, for instance, engaging in negative thoughts and behaviours. The field must be just and equitable to ensure the teammates and the MVP are on the same team fighting against the same opponent. For example, healthcare professionals must have fair conditions to stay on the right team and support the MVP instead of considering them a benchwarmer.

The composition of the MVP's team (social networks and healthcare professionals) and the optimal balance between being the MVP and passing the ball (being capable and accepting support to be capable) varies from person to person and over time. Persons with chronic pain feel valued and supported when the healthcare sector and social networks strengthen their capability by encouraging them to be the MVP and pass the ball. The balancing act between the two is the meaning of lived experiences of support.

[INSERT FIGURE 1 HERE]

Legend: Figure 1. The match against pain. Most Valuable Player/MVP (person with chronic pain) with the ball (pain management), the team (examples of teammates), the opponent (pain), the referee (representing fair conditions), the stadium (life), and the trophy (symbolising the aspiration for a good life despite living with chronic pain).

For peer review only

DISCUSSION

This study applied an interpretative approach in explaining the meaning of support from the healthcare sector and social networks of persons with chronic pain. The findings indicate that, regardless of who is providing the support, the meaning of support when living with chronic pain is to strengthen the individuals' capability and, when the abilities do not seem enough, feel that someone is fighting together with the individual to regain their capability. Previous studies have mainly performed descriptive analyses, focusing on pain management rather than on the meaning of support (13-15). Holtrop et al. (14) found three primary purposes of important relationships in pain management: providing instrumental support, offering inspiration and motivation, and assisting in decision making. Our results are similar but show that support strengthens the participants' capability. In line with the present study Holtrop et al. (14) found that persons with chronic pain wanted to be recognised as persons rather than their condition and that their lives should be seen as no different from others. Meanwhile, they wanted their limitations due to pain to be accepted. Our study also shows that pets could provide this support, which aligns with Bair et al. (13), who demonstrated that pets can be powerful motivators in pain management. The Bair et al. (13) participants relied on support from care managers. Similarly, our results show that support means having someone fight for individuals with chronic pain when their abilities are inadequate.

Acknowledging the patient as an expert and capable person is fundamental to person-centred care (31). The present study clarifies that persons with chronic pain want to be active and recognised as capable, productive partners in care. Accepting support can strengthen their capability, which is also emphasised in person-centred care (31). The

For peer review only

capabilities approach focuses on human development and social justice, recognising that people's capabilities are shaped and formed by environmental and social circumstances (32). A systematic review investigating empirical evidence underpinning the conceptualisations of person-centred care for serious illness found that person-centred care empowers patients *and* their families by providing information and including them in decision-making actions on their daily lives and care (33). Considering these results, person-centred care may be valuable in enhancing capability. It would be worthwhile to explore whether this is the case among persons with chronic pain and their close others.

Methodological considerations

Lindseth and Norberg suggest that phenomenological hermeneutics seeks not to encapsulate the whole truth but to present meanings of a lived phenomenon vis-à-vis interpreting the narrative text (30). Findings are valid if they represent meaning derived from narrated experiences and illuminate something we want to understand (30). One-sided opinions can, however, emerge and conscious validation of the interpretation and analysis become important (30). The hermeneutic spiral, in which pending between understanding (naïve understanding) and explanation (structural analysis), constitutes a reliable approach to validate the findings (30). Discussions between the authors ensured the interpretations were plausible while not being the only possible options.

Additionally, to ensure that the interpretations were reasonable one author (ML) read all the interviews before participating in the analysis. Qualitative samples should be large enough to understand the studied phenomenon but small enough not to hinder qualitative analysis (34). In phenomenology saturation is not used to determine the number of

For peer review only

participants (35); fewer participants are needed if the data are rich (36). The research group decided the collected data sufficed to answer the research question and was not too extensive to capture the meaning of the phenomenon. Most participants in the current study had post-high-school education, were female, and were born in Sweden, which could affect the universality of the findings. Universality is described as an intersubjective understanding of the meaning of lived experiences, meaning that persons can understand the phenomenon better through the findings, even though their situation might not perfectly align with the findings (30). Because all participants had lived with pain for many years, they had broad experiences of support and provided rich narratives. Follow-up questions were used to ensure that the interviewer understood them correctly. Measures were taken to make participants comfortable (confirming they knew they could ask questions, take breaks, etc.) and to encourage sharing their narratives (23). However, Bruce et al. described the chronic "pain journey to acceptance" and that different coping mechanisms are useful depending on where the person is in the journey (37), which might also translate to support. Some participants grew up with pain, which might have affected our findings. However, participants narrated their lived experiences of support as adults while comparing them to their experiences as children rather than merging them. The need to use Zoom™ due to the COVID-19 pandemic might have affected the content of the interviews. Still, video interviews are cost-effective and inclusive (28).

Metaphors were employed in the interpretation process. Ricoeur contends that the metaphor enriches the meaning of a phenomenon through the creative tension of similarities and differences, creating a new understanding (38). The metaphors demonstrate how language can extend to its limits and affect how we understand the world.

For peer review only

CONCLUSIONS

For participants, who lived with chronic pain, support means balancing between being capable (the MVP) and willing to accept support (passing the ball), which aligns with the concept of person-centred care. Our findings may be useful for policymakers, managers, and clinical professionals, may be useful when planning and performing care for persons with chronic pain. Future research should focus on how the healthcare sector can create support to enable persons with chronic pain to be the most valuable players (MVP) while being able to pass the ball to their social networks and the healthcare sector.

CONTRIBUTIONS

VL, SW, IE, and ML planned the study. VL performed the data collection. VL, IE, SW, ML, MS, and VS contributed to the data analysis and interpretation of the findings. VL drafted the manuscript and ML, MS, SW, VS, and IE critically reviewed it and contributed to the final version prior to submission.

ACKNOWLEDGMENTS

The authors gratefully acknowledge the study participants for sharing their narratives. We also appreciate the patient organisations (The Swedish Rheumatism Association, The Swedish Association for Survivors of Accident and Injury, Riksförbundet Ehlers-Danlos Syndrom, and The Swedish National Organization for Young Rheumatics) for facilitating the recruitment.

For peer review only

FUNDING

This work was supported by FORTE grant number [2019-00718] and the University of Gothenburg Centre for Person-Centred Care (GPCC), grant number N/A.

COMPETING INTEREST

None declared.

For peer review only

ETHICS APPROVAL

Ethical approval for this study was granted by the Swedish Ethical Review Authority (Reg nr 2020-02491). The study was conducted according to the ethical principles outlined by the Declaration of Helsinki (29).

DATA AVAILABILITY STATEMENT

No additional data are available.

CREATIVE COMMONS LICENCE, CC-BY

For the purpose of Open Access, the authors have applied a CC BY public copyright licence to any Author Accepted Manuscript (AAM) version arising from this submission.

REFERENCES

1. International Association for the Study of Pain I. IASP Revises Its Definition of Pain for the First Time Since 1979 2020 [Available from: https://www.iasp-pain.org/wp-content/uploads/2022/04/revised-definition-flysheet_R2.pdf.
2. Treede RD, Rief W, Barke A, Aziz Q, Bennett MI, Benoliel R, et al. Chronic pain as a symptom or a disease: the IASP Classification of Chronic Pain for the International Classification of Diseases (ICD-11). Pain. 2019;160(1):19-27.
3. Breivik H, Collett B, Ventafridda V, Cohen R, Gallacher D. Survey of chronic pain in Europe: prevalence, impact on daily life, and treatment. European journal of pain (London, England). 2006;10(4):287-333.
4. Dahlhamer J, Lucas J, Zelaya C, Nahin R, Mackey S, DeBar L, et al. Prevalence of Chronic Pain and High-Impact Chronic Pain Among Adults - United States, 2016. MMWR Morbidity and mortality weekly report. 2018;67(36):1001-6.
5. Foley HE, Knight JC, Ploughman M, Asghari S, Audas R. Association of chronic pain with comorbidities and health care utilisation: a retrospective cohort study using health administrative data. Pain. 2021;162(11):2737-49.
6. O'Brien T, Breivik H. The impact of chronic pain-European patients' perspective over 12 months. Scandinavian journal of pain. 2012;3(1):23-9.
7. Fredheim OM, Kaasa S, Fayers P, Saltnes T, Jordhøy M, Borchgrevink PC. Chronic non-malignant pain patients report as poor health-related quality of life as palliative cancer patients. Acta anaesthesiologica Scandinavica. 2008;52(1):143-8.
8. Lundin Å, Ekman I, Wallström S, Andréll P, Lundberg M. Suffering out of sight but not out of mind - interpreting experiences of sick leave due to chronic pain in a community setting: a qualitative study. BMJ open. 2023;13(4):e066617.
9. Häuser W, Wolfe F, Henningsen P, Schmutzer G, Brähler E, Hinz A. Untying chronic pain: prevalence and societal burden of chronic pain stages in the general population - a cross-sectional survey. BMC public health. 2014;14:352.
10. de Sola H, Salazar A, Dueñas M, Ojeda B, Failde I. Nationwide cross-sectional study of the impact of chronic pain on an individual's employment: relationship with the family and the social support. BMJ open. 2016;6(12):e012246.
11. Gustavsson A, Bjorkman J, Ljungcrantz C, Rhodin A, Rivano-Fischer M, Sjolund KF, et al. Socio-economic burden of patients with a diagnosis related to chronic pain--register data of 840,000 Swedish patients. European journal of pain (London, England). 2012;16(2):289-99.
12. Devan H, Hale L, Hempel D, Saipe B, Perry MA. What Works and Does Not Work in a Self-Management Intervention for People With Chronic Pain? Qualitative Systematic Review and Meta-Synthesis. Physical therapy. 2018;98(5):381-97.
13. Bair MJ, Matthias MS, Nyland KA, Huffman MA, Stubbs DL, Kroenke K, et al. Barriers and facilitators to chronic pain self-management: a qualitative study of primary care patients with comorbid musculoskeletal pain and depression. Pain medicine (Malden, Mass). 2009;10(7):1280-90.
14. Holtrop JS, Fisher M, Martinez DE, Simpson M, Awadallah NS, Nease DE, Jr., et al. What Works for Managing Chronic Pain: An Appreciative Inquiry Qualitative Analysis. Journal of primary care & community health. 2019;10:2150132719885286.
15. Franklin ZC, Smith NC, Fowler NE. A qualitative investigation of factors that matter to individuals in the pain management process. Disability and rehabilitation. 2016;38(19):1934-42.
16. Pester BD, Tankha H, Caño A, Tong S, Grekin E, Bruinsma J, et al. Facing Pain Together: A Randomized Controlled Trial of the Effects of Facebook Support Groups on Adults With Chronic Pain. The journal of pain. 2022;23(12):2121-34.
17. Matthias MS, McGuire AB, Kukla M, Daggy J, Myers LJ, Bair MJ. A brief peer support intervention for veterans with chronic musculoskeletal pain: a pilot study of feasibility and effectiveness. Pain medicine (Malden, Mass). 2015;16(1):81-7.

For peer review only

18. van Lankveld W, van Helmond T, Näring G, de Rooij DJ, van den Hoogen F. Partner participation in cognitive-behavioral self-management group treatment for patients with rheumatoid arthritis. The Journal of rheumatology. 2004;31(9):1738-45.
19. Riemsma RP, Taal E, Rasker JJ. Group education for patients with rheumatoid arthritis and their partners. Arthritis and rheumatism. 2003;49(4):556-66.
20. Gatchel RJ, Peng YB, Peters ML, Fuchs PN, Turk DC. The biopsychosocial approach to chronic pain: scientific advances and future directions. Psychological bulletin. 2007;133(4):581-624.
21. Cohen SP, Vase L, Hooten WM. Chronic pain: an update on burden, best practices, and new advances. Lancet (London, England). 2021;397(10289):2082-97.
22. Hadi MA, Alldred DP, Briggs M, Marczewski K, Closs SJ. 'Treated as a number, not treated as a person': a qualitative exploration of the perceived barriers to effective pain management of patients with chronic pain. BMJ open. 2017;7(6):e016454.
23. Lindseth A, Norberg A. A phenomenological hermeneutical method for researching lived experience. Scandinavian journal of caring sciences. 2004;18(2):145-53.
24. O'Brien BC, Harris IB, Beckman TJ, Reed DA, Cook DA. Standards for reporting qualitative research: a synthesis of recommendations. Academic medicine : journal of the Association of American Medical Colleges. 2014;89(9):1245-51.
25. Perrot S, Cohen M, Barke A, Korwisi B, Rief W, Treede RD. The IASP classification of chronic pain for ICD-11: chronic secondary musculoskeletal pain. Pain. 2019;160(1):77-82.
26. Palinkas LA, Horwitz SM, Green CA, Wisdom JP, Duan N, Hoagwood K. Purposeful Sampling for Qualitative Data Collection and Analysis in Mixed Method Implementation Research. Administration and policy in mental health. 2015;42(5):533-44.
27. Brinkmann S, Kvale S. InterViews - Learning the Craft of Qualitative Reserach Interviewing. 3rd ed. Los Angeles: SAGE Publications, Inc.; 2015.
28. Saarijärvi M, Bratt EL. When face-to-face interviews are not possible: tips and tricks for video, telephone, online chat, and email interviews in qualitative research. European journal of cardiovascular nursing : journal of the Working Group on Cardiovascular Nursing of the European Society of Cardiology. 2021;20(4):392-6.
29. World Medical Association Declaration of Helsinki: ethical principles for medical research involving human subjects. Jama. 2013;310(20):2191-4.
30. Lindseth A, Norberg A. Elucidating the meaning of life world phenomena. A phenomenological hermeneutical method for researching lived experience. Scandinavian journal of caring sciences. 2022;36(3):883-90.
31. Ekman I. Practising the ethics of person-centred care balancing ethical conviction and moral obligations. Nursing philosophy : an international journal for healthcare professionals. 2022;23(3):e12382.
32. Entwistle VA, Watt IS. Treating patients as persons: a capabilities approach to support delivery of person-centered care. The American journal of bioethics : AJOB. 2013;13(8):29-39.
33. Giusti A, Nkhoma K, Petrus R, Petersen I, Gwyther L, Farrant L, et al. The empirical evidence underpinning the concept and practice of person-centred care for serious illness: a systematic review. BMJ global health. 2020;5(12).
34. Sandelowski M. Sample size in qualitative research. Research in nursing & health. 1995;18(2):179-83.
35. van Manen M, Higgins I, van der Riet P. A conversation with Max van Manen on phenomenology in its original sense. Nursing & health sciences. 2016;18(1):4-7.
36. Morse JM. Determining Sample Size. Qualitative health research. 2000;10(1):3-5.
37. Bruce M, Lopatina E, Hodge J, Moffat K, Khan S, Pyle P, et al. Understanding the chronic pain journey and coping strategies that patients use to manage their chronic pain: a qualitative, patient-led, Canadian study. BMJ open. 2023;13(7):e072048.
38. Ricoeur P. The rule of metaphor. Abingdon: Routledge Press; 2003.

For peer review only

For peer review only

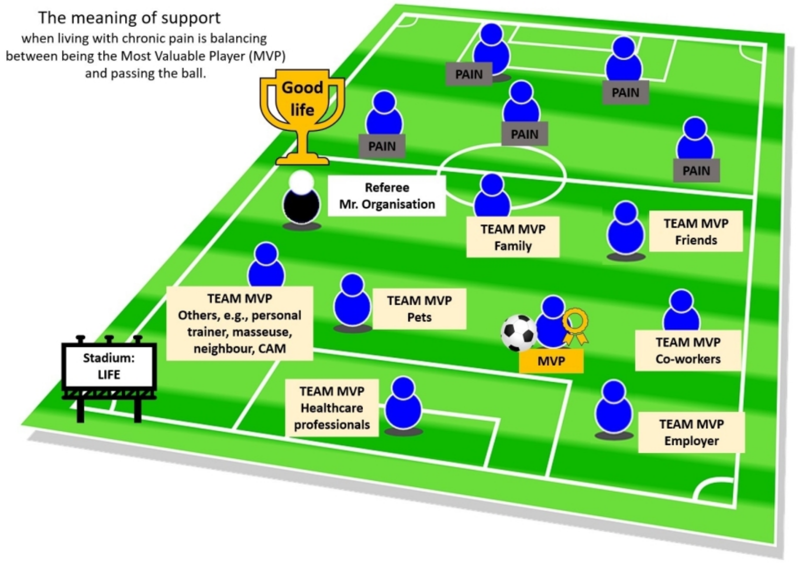

Figure 1. The match against pain. Most Valuable Player/MVP (person with chronic pain) with the ball (pain management), the team (examples of teammates), the opponent (pain), the referee (representing fair conditions), the stadium (life), and the trophy (symbolising the aspiration for a good life despite living with chronic pain).

162x91mm (300 x 300 DPI)

Pre-understanding

I believe persons with chronic pain often feel as if they have been treated poorly in encounters with healthcare professionals. I think they perceive that their pain is not taken seriously and that they are not being believed. I also think they perceive they are not being given the required help and struggle to get help. I believe they mostly have been in contact with medical doctors.

I believe they experience that their social network is not being invited to participate in their care. I think they feel support from their social network without including the network within their care. I do not know how they perceive support, but I can imagine that the employer, family, and friends are significant and their relationships are often impacted by pain. People with chronic pain want to involve their social network if healthcare professionals ask them.

For peer review only

Interview guide

The aim is to elucidate the meaning of lived experiences of support from the social network and healthcare sector in persons with chronic pain.

Research questions

How do persons with chronic pain experience support from the healthcare sector?

How do persons with chronic pain experience support from their social network outside the healthcare sector?

How do persons with chronic pain perceive involving their social network in their care and rehabilitation?

Support within the healthcare sector

Do you want to tell me when you first contacted the healthcare sector regarding your pain?

How have you perceived the help you've gotten from the healthcare sector since then?

- What did you feel when you did/did not receive that help?
- How has the help you've received from the healthcare sector made a difference to you?

What kind of help do you feel you would have liked to have but have not gotten from the healthcare sector?

- Are there any symptoms you would have liked to receive more help with?
- How did you feel when you received/did not receive that help?

For peer review only

How would you like the healthcare sector to help you?

Support from the social network

Which persons outside of the healthcare sector have been important to you regarding your pain?

- How has contact with those people helped you?
- What kind of difference has that contact made for you?

Are there others outside the healthcare sector who have been important to you regarding your pain?

- How has contact with these people helped you?
- What difference has the contact with these persons made for you?

Are there any persons outside the healthcare sector you would have liked to receive more support from?

- Do you want to tell me more about this?
- What did you feel when you did not receive that support?

Involvement of the social network in care

How have persons from outside the healthcare sector been involved in your care? (for instance, next of kin).

- In which parts of the care have they been involved?
- How did it come about that they got involved?

For peer review only

- Who initiated the involvement?

How has it worked to have them involved in your care?

- How have you experienced it?

How do you feel about involving persons outside of the healthcare sector in your care?

- In which situations would you like to involve persons from outside the healthcare sector in your care?

What would facilitate involving other persons in your care?

How do you perceive the healthcare sector's attitude towards involving other persons?

Concluding

Is there anything that we've discussed that you would like to talk more about or something we haven't addressed that you would like to add?

For peer review only

Standards for Reporting Qualitative Research (SRQR)*

http://www.equator-network.org/reporting-guidelines/srqr/

Title and abstract

	Page/line no(s).
**Title** - Concise description of the nature and topic of the study Identifying the study as qualitative or indicating the approach (e.g., ethnography, grounded theory) or data collection methods (e.g., interview, focus group) is recommended	p1 / 1-3
**Abstract** - Summary of key elements of the study using the abstract format of the intended publication; typically includes background, purpose, methods, results, and conclusions	p3 / 1-17

Introduction

**Problem formulation** - Description and significance of the problem/phenomenon studied; review of relevant theory and empirical work; problem statement	p5 / 1-23<br>p6 / 1-14
**Purpose or research question** - Purpose of the study and specific objectives or questions	p6 / 25-16

Methods

**Qualitative approach and research paradigm** - Qualitative approach (e.g., ethnography, grounded theory, case study, phenomenology, narrative research) and guiding theory if appropriate; identifying the research paradigm (e.g., postpositivist, constructivist/ interpretivist) is also recommended; rationale**	P6 / 19-21<br>P7 / 1
**Researcher characteristics and reflexivity** - Researchers' characteristics that may influence the research, including personal attributes, qualifications/experience, relationship with participants, assumptions, and/or presuppositions; potential or actual interaction between researchers' characteristics and the research questions, approach, methods, results, and/or transferability	p8 / 7-12
**Context** - Setting/site and salient contextual factors; rationale**	p7 / 5-8
**Sampling strategy** - How and why research participants, documents, or events were selected; criteria for deciding when no further sampling was necessary (e.g., sampling saturation); rationale**	p7/ 8-23<br>p8 / 1-6
**Ethical issues pertaining to human subjects** - Documentation of approval by an appropriate ethics review board and participant consent, or explanation for lack thereof; other confidentiality and data security issues	p9 /14-21
**Data collection methods** - Types of data collected; details of data collection procedures including (as appropriate) start and stop dates of data collection and analysis, iterative process, triangulation of sources/methods, and modification of procedures in response to evolving study findings; rationale**	P9 / 1-7

For peer review only

For peer review only - http://bmjopen.bmj.com/site/about/guidelines.xhtml

**Data collection instruments and technologies** - Description of instruments (e.g., interview guides, questionnaires) and devices (e.g., audio recorders) used for data collection; if/how the instrument(s) changed over the course of the study	p8 / 14-23<br>p9 / 1, 4-5
**Units of study** - Number and relevant characteristics of participants, documents, or events included in the study; level of participation (could be reported in results)	P12 / 14-18<br>P13 / 1<br>P14/1
**Data processing** - Methods for processing data prior to and during analysis, including transcription, data entry, data management and security, verification of data integrity, data coding, and anonymization/de-identification of excerpts	P9 / 4-5, 19-21<br>P10 / 14
**Data analysis** - Process by which inferences, themes, etc., were identified and developed, including the researchers involved in data analysis; usually references a specific paradigm or approach; rationale**	P10 / 1-21<br>p11 / 1-8<br>P12 / 1-11
**Techniques to enhance trustworthiness** - Techniques to enhance trustworthiness and credibility of data analysis (e.g., member checking, audit trail, triangulation); rationale**	P10 / 5-7, 17-21<br>P11 / 1-2, 7-8<br>P12/1-11

Results/findings

**Synthesis and interpretation** - Main findings (e.g., interpretations, inferences, and themes); might include development of a theory or model, or integration with prior research or theory	P12/13-18<br>P13/1<br>P14/1-14<br>P15/1-20<br>P16/1-15<br>P17/1-22<br>P18/1-23<br>P19/1-22<br>P20/1-21<br>P21/1-23<br>P22/1-23<br>P23/1-23<br>P24/1-23
**Links to empirical data** - Evidence (e.g., quotes, field notes, text excerpts, photographs) to substantiate analytic findings	P17/4-9, 16-18<br>P18/1-4, 20-23<br>P19/5-7<br>P20/5-16<br>P21/9-11, 20-23<br>P22/1, 8-12, 21-23<br>P23/1-4

Discussion

**Integration with prior work, implications, transferability, and contribution(s) to the field -** Short summary of main findings; explanation of how findings and conclusions connect to, support, elaborate on, or challenge conclusions of earlier scholarship; discussion of scope of application/generalizability; identification of unique contribution(s) to scholarship in a discipline or field	P25/2-23<br>P26/1-8
**Limitations** - Trustworthiness and limitations of findings	p26 / 10-23<br>p27 /1-23

For peer review only - http://bmjopen.bmj.com/site/about/guidelines.xhtml

For peer review only

Other

**Conflicts of interest** - Potential sources of influence or perceived influence on study conduct and conclusions; how these were managed	p29/5-6
**Funding** - Sources of funding and other support; role of funders in data collection, interpretation, and reporting	p29/1-3

*The authors created the SRQR by searching the literature to identify guidelines, reporting standards, and critical appraisal criteria for qualitative research; reviewing the reference lists of retrieved sources; and contacting experts to gain feedback. The SRQR aims to improve the transparency of all aspects of qualitative research by providing clear standards for reporting qualitative research.

**The rationale should briefly discuss the justification for choosing that theory, approach, method, or technique rather than other options available, the assumptions and limitations implicit in those choices, and how those choices influence study conclusions and transferability. As appropriate, the rationale for several items might be discussed together.

Reference:
O'Brien BC, Harris IB, Beckman TJ, Reed DA, Cook DA. **Standards for reporting qualitative research: a synthesis of recommendations.** *Academic Medicine*, Vol. 89, No. 9 / Sept 2014 DOI: 10.1097/ACM.0000000000000388

For peer review only
